# Enhancing Spatial Transcriptomics via Spatially Constrained Matrix Decomposition with EDGES

**DOI:** 10.1002/advs.202508346

**Published:** 2025-08-21

**Authors:** Jinyue Zhao, Jiating Yu, Yuqing Cao, Fan Yuan, Ling‐Yun Wu, Duanchen Sun

**Affiliations:** ^1^ School of Mathematics Shandong University Jinan 250100 China; ^2^ School of Mathematics and Statistics Nanjing University of Information Science & Technology Nanjing 210044 China; ^3^ School of Mathematics and Information Science Yantai University Yantai 264005 China; ^4^ State Key Laboratory of Mathematical Sciences Academy of Mathematics and Systems Science Chinese Academy of Sciences Beijing 100190 China; ^5^ School of Mathematical Sciences University of Chinese Academy of Sciences Beijing 100049 China; ^6^ Shandong Key Laboratory of Cancer Digital Medicine Jinan 250033 China

**Keywords:** data integration, denoising, matrix decomposition, spatial transcriptomics

## Abstract

Spatial transcriptomics (ST) technologies revolutionize biomedical research by providing unprecedented insights into tissue architecture and disease mechanisms. While imaging‐based ST technologies achieve single‐cell spatial resolution, they face inherent limitations in gene detection capacity and measurement accuracy of expression profiles. Although computational approaches make notable progress, current methods remain challenged by insufficient integration of spatial context and systematic biases toward the single‐cell RNA sequencing distribution. To address these limitations, EDGES is developed a spatially constrained non‐negative matrix factorization framework that simultaneously predicts undetected gene expression and denoises measured transcriptional profiles. EDGES incorporates spatial information through graph Laplacian regularization while synergistically integrating cellular representations with gene‐specific representations, thereby ensuring that the predicted gene expression aligns closely with the real ST distribution. Comprehensive evaluations demonstrate that EDGES achieves superior predictive performance and outperforms existing denoising methods. The framework's versatility further facilitates the identification of novel biological markers and spatially resolved expression patterns. With its innovative design, EDGES provides an advanced tool to enhance the reliability of the imaging‐based ST data, facilitating more accurate and biologically meaningful interpretation of downstream discoveries.

## Graphical abstract

Imaging‐based spatial transcriptomics technologies are currently limited by their restricted gene detection capacity and low measurement accuracy. Moreover, the insufficient integration of spatial context and single‐cell reference data poses a significant challenge for comprehensive data analysis. EDGES addresses these issues by employing a spatially constrained non‐negative matrix factorization framework, effectively enhancing the quality of imaging‐based spatial transcriptomics data.

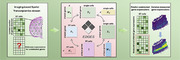

## 1 Introduction

Single‐cell RNA sequencing (scRNA‐seq) technologies have significantly advanced our understanding of cellular heterogeneity and complex biological systems by enabling high‐resolution gene expression profiling at the single‐cell level.^[^ ^1^, ^2^ ^]^ However, these technologies inherently dissociate cells from their native spatial context, resulting in the loss of crucial spatial information.^[^ ^3^, ^4^ ^]^ To address this limitation, spatial transcriptomics (ST) has emerged as a powerful approach that preserves spatial localization while capturing gene expression profiles, thereby enabling more comprehensive insights into cellular communication, tissue organization, and disease pathogenesis.^[^ ^3^, ^4^, ^5^ ^]^

The current ST technologies can be broadly classified into two main categories: sequencing‐based and imaging‐based. Sequencing‐based technologies, such as 10X Visium,^[^ ^6^ ^]^ Slide‐seq,^[^ ^7^ ^]^ and Stereo‐seq,^[^ ^8^ ^]^ offer transcriptome‐wide throughput but are constrained by relatively low spatial resolution.^[^ ^3^, ^4^, ^5^, ^9^, ^10^ ^]^ In contrast, imaging‐based technologies, including osmFISH,^[^ ^11^ ^]^ MERFISH,^[^ ^12^ ^]^ seqFISH,^[^ ^13^ ^]^ and STARmap,^[^ ^14^ ^]^ achieve single‐cell or even subcellular resolution.^[^ ^3^, ^12^, ^13^, ^14^ ^]^ However, they are typically limited by the number of genes that can be detected. In addition to these trade‐offs, both types of ST technologies suffer from varying degrees of technical noise, which complicates downstream analyses and hinders the accurate interpretation of spatial gene expression patterns.

To enhance the quality of imaging‐based ST data, one effective approach is to incorporate comprehensive gene expression information from reference scRNA‐seq datasets. Building on this idea, a variety of computational methods have been proposed to predict the expressions for the undetected genes. For example, SpaGE,^[^ ^15^ ^]^ stPlus,^[^ ^16^ ^]^ and iSpatial^[^ ^17^ ^]^ adopt a joint embedding strategy by constructing a shared low‐dimensional latent space between ST and scRNA‐seq data, followed by a k‐nearest neighbor (KNN)‐based aggregation to infer undetected gene expression. In contrast, Tangram^[^ ^18^ ^]^ uses probabilistic similarity matrices to spatially align scRNA‐seq profiles with ST data, enabling gene expression prediction through linear aggregation. ENGEP^[^ ^19^ ^]^ is an ensemble learning‐based tool to achieve a more consistent and accurate prediction, while SpatialScope^[^ ^20^ ^]^ employs deep generative models to learn the distribution of gene expression from the scRNA‐seq data, allowing more in‐depth and informative downstream analyses at single‐cell resolution.

While the current methods that predict the expressions for undetected genes have made significant advances, they still face several critical limitations. A major concern is that many approaches insufficiently integrate spatial information, treating gene expression enhancement as a purely predictive task but neglecting the underlying tissue architecture. Besides, the heavy reliance on scRNA‐seq data may introduce systematic biases, potentially distorting spatially relevant gene expression patterns. Furthermore, most methods focus primarily on predicting undetected genes while overlooking the technical noise inherent in ST data, which can significantly compromise the accuracy and robustness of downstream analyses.

To overcome these challenges, we developed EDGES, a spatially constrained non‐negative matrix factorization (NMF) framework^[^ ^21^ ^]^ that simultaneously predicts undetected gene expression and denoises measured transcriptional profiles. EDGES incorporates spatial information through graph Laplacian regularization while synergistically integrating cellular representations with gene‐specific representations, thereby ensuring that the predicted gene expression aligns closely with the real ST distributions. By jointly learning interpretable representations for both genes and cells, EDGES achieves superior predictive performance and outperforms existing denoising methods across a variety of datasets. The framework's versatility further facilitates the identification of novel biological markers and spatially resolved expression patterns. Our studies demonstrated that EDGES can serve as an advanced tool to enhance the reliability of the imaging‐based ST data, facilitating more accurate and biologically meaningful interpretation of downstream discoveries.

## 2 Results

### 2.1 Overview of EDGES

**Figure 1:**
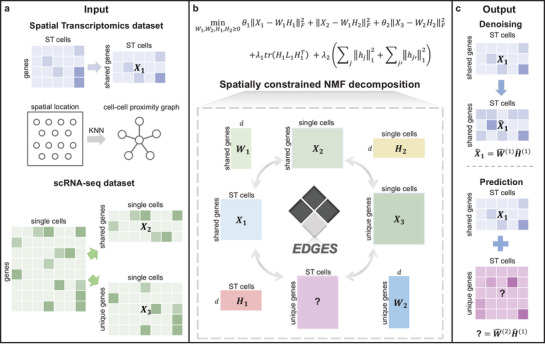
Overview of EDGES. a) The inputs of EDGES consist of ST data and a reference scRNA‐seq data. EDGES partitions the ST and scRNA‐seq data into *X*_1_, *X*_2_, and *X*_3_ according to the shared genes and constructs, a cell–cell proximity graph based on the spatial coordinates. b) The optimization problem of EDGES. EDGES employs a spatially constrained NMF decomposition strategy to obtain joint low‐dimensional representations for ST and scRNA‐seq data. c) The outputs of EDGES include a denoised ST gene expression profile for measured genes and the predicted expressions for undetected genes.

The workflow of EDGES is illustrated in **Figure** **1**. The inputs of EDGES contain ST data and corresponding reference scRNA‐seq data. EDGES extracts the shared gene expression matrix from the ST data (denoted as *X*_1_) and partitions the scRNA‐seq data into shared (denoted as *X*_2_) and unique gene expression components (denoted as *X*_3_) (Figure 1a). To ensure consistent low‐dimensional representations across modalities, EDGES employs a mutually coupled decomposition strategy based on NMF (Methods). Spatial information is further incorporated through a graph‐based regularization term, enabling the preservation of the underlying tissue architecture (Figure 1b). After solving the optimization problem, EDGES produces denoised ST gene expression profiles and the predicted expressions for undetected genes (Figure 1c). These outputs can be used to identify novel biological markers and spatially resolved expression patterns in downstream analyses.

### 2.2 EDGES has Superior Predictive Performance Than Existing Tools in Validating Measured Spatial Expressions

**Figure 2:**
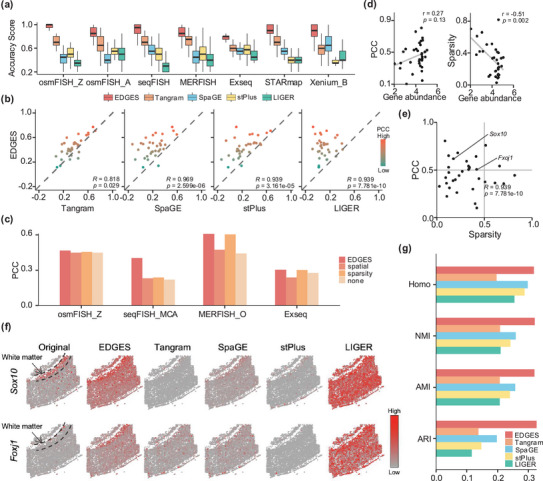
Benchmarking results on validating measured spatial expressions. a) Boxplots show the accuracy scores of each method on seven datasets (*n* = 33, 33, 351, 268, 42, 1020, and 313 in each boxplot). The box plot center line and the box limits represent the median value and upper and lower quartiles, respectively. Box whiskers indicate the largest and smallest values no more than 1.5 times the interquartile range from the limits. b) Scatter plots show the prediction performance comparisons between EDGES and other methods (*n* = 33 in each plot). *R* is the proportion of genes with higher Pearson Correlation Coefficient (PCC) in EDGES, and the statistical *p*‐value was determined by the Wilcoxon rank‐sum test. c) Barplot illustrates the PCC derived from ablation experiments across four datasets: full model (EDGES), model with spatial manifold regularization only (spatial), model with sparsity regularization only (sparsity), and model without regularizations (none). d) Scatter plots show the relationships among gene abundance score, PCC, and gene sparsity (*n* = 33 in each plot). The *r* value represents the correlation coefficient, and the statistical *p* value was determined by the Student’*s t*‐test. The linear regression lines represent the trends between the corresponding variables. e) Scatter plot shows the relationship between PCC and gene sparsity (*n* = 33 in each plot). f) Visualizations of the raw spatial expressions and the predicted expressions using EDGES and other methods. g) Barplot shows the clustering performances of five methods across four metrics.

We first benchmarked the predictive performance of EDGES against other state‐of‐the‐art methods by validating measured spatial expression patterns. To this end, we selected seven imaging‐based technologies (osmFISH, seqFISH, MERFISH, Exseq, STARmap, Xenium, and seqFISH+) that detected genes ranging from tens to thousands and conducted a series of cross‐validation experiments (Methods). EDGES outperformed other methods with the highest Accuracy Score (AS) across all twelve applications (**Figure** **2**a; Figures S1 and S2a, Supporting Information). Specifically, EDGES improved the average AS by 37.31%, 32.18%, 28.48%, 15.80%, 28.40%, 28.57%, and 50.21% compared to the second‐best approach, Tangram (Figure 2a). For example, when integrating the osmFISH ST data with Zeisel's reference scRNA‐seq data (osmFISH_Z), over 81.8% of genes predicted by EDGES exhibited spatial expressions more consistent with the ground truth than those predicted by Tangram (Figure 2b, Wilcoxon rank‐sum test *p* = 0.029). This trend was even more pronounced when compared to SpaGE, stPlus, and LIGER (Figure 2b). When directly comparing EDGES with the results reported in,^[^ ^10^ ^]^ EDGES also showed a comparable predictive performance and maintained its advantage even when the second‐best approach, Tangram, was aligned using identical parameters as in,^[^ ^10^ ^]^ providing additional confidence that EDGES matches or outperforms previous state‐of‐the‐art approaches (Table S3 and Figure S3, Supporting Information). Furthermore, ablation experiments across multiple datasets confirmed that both the spatial and sparsity regularization terms contributed to EDGES's superior predictive performance (Figure 2c).

Utilizing the flexible NMF framework in EDGES, we defined an abundance score for each gene based on the decomposed low‐dimensional representations (Methods). These gene abundance scores exhibited a positive correlation with predictive performances and a negative correlation with the gene sparsity levels (Figure 2d). Interestingly, no significant correlation was observed between predictive performance and sparsity level within osmFISH_Z (Figure 2e), suggesting that the learned representations effectively captured biologically meaningful information relevant to gene expression patterns. To further validate EDGES, we visualized two genes with distinct sparsity levels and observed that their predicted spatial expression patterns closely matched the original measurements (Figure 2f). For example, EDGES accurately reconstructed the spatial distribution of *Foxj1*, a gene specifically expressed in the white matter layer, whereas other competing methods failed to recover its pattern.

We further assessed the predictive performance from a cell‐level perspective. Since unsupervised clustering plays a critical role in deciphering cellular heterogeneity, we employed widely used clustering evaluation metrics to compare the predictive performance of different methods. EDGES achieved the highest scores in Homogeneity (Homo), Normalized Mutual Information (NMI), Adjusted Mutual Information (AMI), and Adjusted Rand Index (ARI) in the osmFISH_Z dataset. In contrast, Tangram exhibited less satisfactory clustering performances at the cell level (Figure 2g). Besides, EDGES showed strong predictive performance at the cell level across all benchmarking datasets (Figure S2b, Supporting Information). Collectively, these findings demonstrated the superior predictive performances of EDGES and its effectiveness in validating measured spatial expression patterns.

### 2.3 EDGES Accurately Predicts Biologically Meaningful Undetected Genes

**Figure 3:**
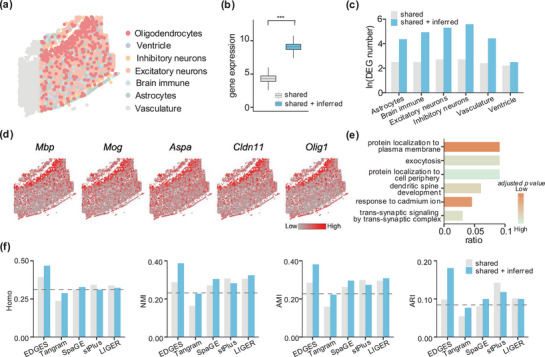
EDGES accurately predicts biologically meaningful undetected genes. a) Manual annotation of cell types of the mouse cortex osmFISH dataset. b) Boxplot shows the total gene expression per cell in the unprocessed and EDGES‐processed data (*n* = 6471 in each boxplot). The box plot center line and the box limits represent the median value and upper and lower quartiles, respectively. Box whiskers indicate the largest and smallest values no more than 1.5 times the interquartile range from the limits. The statistical *p*‐value was determined by the Wilcoxon rank‐sum test with *** representing *p* less than 2.2e‐16. c) Barplot shows the number of DEGs between oligodendrocytes and other cell types identified from the unprocessed and EDGES‐processed data. d) Visualizations of upregulated DEGs specific to oligodendrocytes. e) Barplot shows the functional enrichment of upregulated DEGs specific to oligodendrocytes. f) Barplots show the clustering performance of each method using the shared genes and the shared plus inferred genes. The dashed line indicates the clustering performance based on the original gene expression profiles from the osmFISH dataset.

Next, we quantitatively evaluated the predictive capability of EDGES for undetected genes. To this end, we applied EDGES to an ST dataset with known cell type annotations (**Figure** **3**a). While the original osmFISH data contained only 33 genes, EDGES inferred the expression of 2000 additional genes by integrating a reference scRNA‐seq dataset, substantially expanding the transcriptional landscape of the ST data (Figure 3b, Wilcoxon rank‐sum test *p* < 2.2e‐16).

Benefiting from EDGES, we identified a larger set of differentially expressed genes (DEGs) across various cell types using the edgeR^[^ ^22^ ^]^ (Figure 3c; Figure S4, Supporting Information). Taking oligodendrocytes as an example, which exhibit a distinct band‐like distribution, EDGES detected 121 additional upregulated DEGs specific to this cell type. Among these, known oligodendrocyte marker genes, including *Mbp*, *Mog*, *Aspa*, *Cldn11*, and *Olig1* ^[^ ^23^, ^24^, ^25^, ^26^, ^27^, ^28^, ^29^, ^30^, ^31^ ^]^ were accurately predicted and localized to the oligodendrocyte region (Figure 3d). Functional enrichment analysis of these newly identified upregulated DEGs highlighted their associations with exocytosis, a critical process for myelin release around neurons (Figure 3e; Table S4, Supporting Information). Beyond oligodendrocytes, EDGES also uncovered upregulated DEGs and their functional associations in other cell types, with spatial distributions closely aligning with their respective cell type regions (Figure S5 and Table S4, Supporting Information).

We further assessed whether the inferred gene expression profiles could enhance cell clustering. Using known cell types as the ground truth, we compared the clustering performance of EDGES with that of shared gene expression and four representative methods: Tangram, SpaGE, stPlus, and LIGER. When using only the shared genes, EDGES exhibited a comparable clustering performance. However, upon incorporating the inferred undetected genes, EDGES achieved the most accurate characterization of cellular heterogeneity, showing the greatest improvement across all evaluated metrics (Figure 3f). Notably, EDGES attained the highest Homo scores for both shared genes and all genes, underscoring its superior intra‐clustering consistency. In contrast, stPlus and Tangram showed inferior clustering performances. The clustering quality of stPlus further declined when incorporating predicted transcriptomes, whereas Tangram performed even worse than the clustering performance based on the original gene expression profiles from the osmFISH dataset. (Figure 3f). Additionally, Uniform Manifold Approximation and Projection (UMAP) visualizations of cell clustering across different methods revealed that EDGES produced results most closely resembling the actual spatial distribution of cells (Figure S6, Supporting Information).

In summary, these pieces of evidence demonstrated the effectiveness of EDGES in predicting biologically meaningful undetected genes. EDGES enables a more comprehensive characterization of cellular heterogeneity by identifying cell‐type‐specific DEGs and improving clustering performance.

### 2.4 EDGES Effectively Denoises the Measured Gene Expression Profiles

**Figure 4:**
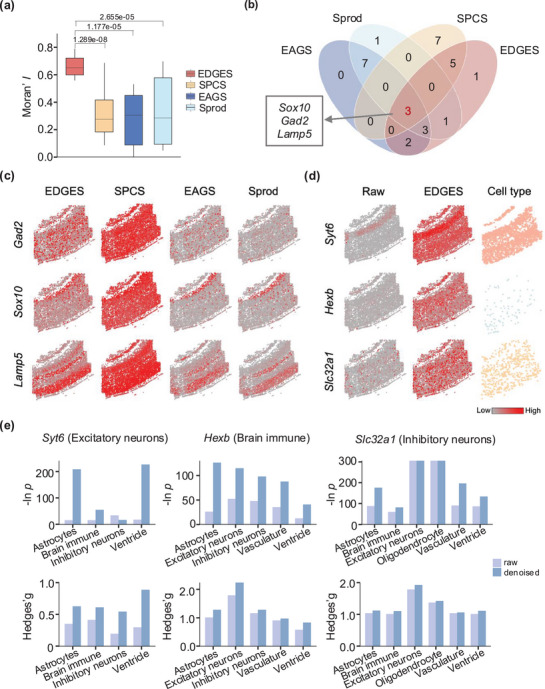
EDGES effectively denoises the measured gene expression profiles of the osmFISH dataset. a) Boxplot shows the quality of the resulting SVGs using the denoised matrices generated by different denoising methods (*n* = 15 in each boxplot). The box plot center line and the box limits represent the median value and upper and lower quartiles, respectively. Box whiskers indicate the largest and smallest values no more than 1.5 times the interquartile range from the limits. The statistical *p*‐value was determined by the Wilcoxon rank‐sum test. b) The Venn diagram shows the overlapped SVGs across different denoising methods. c) Visualizations of specific SVGs with the corresponding expressions denoised by different denoising methods. d) Visualizations of selected marker genes for specific cell types. e) Barplots show the statistical *p*‐values and Hedges'g values between specific and other cell types before and after EDGES denoising.

Leveraging the mutually coupled decomposition strategy, EDGES can denoise the original measured gene expression profiles. To assess its denoising efficacy, we applied the same spatially variable gene (SVG) identification algorithm, Hotspot,^[^ ^32^ ^]^ to the denoised gene expression profiles generated by different denoising methods and compared the quality of the resulting SVGs (Methods). Specifically, we employed Moran's *I* ^[^ ^33^ ^]^ to evaluate the spatial coherence of SVGs and benchmarked EDGES against SPCS,^[^ ^34^ ^]^ EAGS,^[^ ^35^ ^]^ and Sprod.^[^ ^36^ ^]^ As expected, SVGs identified from EDGES‐denoised data exhibited significantly higher spatial coherence than those obtained from other methods, with the average Moran's *I* increase of 0.34, 0.38, and 0.31 compared to SPCS, EAGS, and Sprod, respectively (**Figure** **4**a; Figure S7, Supporting Information, Wilcoxon rank‐sum test *p* < 0.01). Besides, three SVGs were consistently detected across the denoised datasets from all methods (Figure 4b). Visualization of these overlapping SVGs revealed that EDGES generated expression patterns with stronger spatial aggregations compared to other methods (Figure 4c). The robustness of EDGES was further validated using an alternative SVG detection tool, SPARK,^[^ ^37^ ^]^ which also demonstrated the superior denoising performance of EDGES (Figure S8, Supporting Information).

Next, we validated the denoising performance of EDGES from the perspective of marker genes. Compared to the raw data, EDGES‐denoised gene expression profiles better captured the inherent heterogeneity among cell types. Notably, the expression patterns of key marker genes, including the excitatory neuron marker *Syt6*, the brain immune marker *Hexb*, and the inhibitory neuron marker *Slc32a1*, aligned more closely with their expected cell‐type distributions after denoising (Figure 4d). Furthermore, these marker genes exhibited significantly lower *p* values and higher Hedges'g values compared to the raw data, indicating a more pronounced distinction between cell types (Figure 4e). This suggests that EDGES not only reduces noise but also effectively preserves biologically meaningful signals, enhancing the interpretability of ST data.

The above observations demonstrated the effectiveness of EDGES in denoising ST data, thereby facilitating more accurate downstream analyses of gene expression profiles and cellular heterogeneity.

### 2.5 EDGES Advances Spatial Proteomics Across Applications in Human Bone Marrow

**Figure 5:**
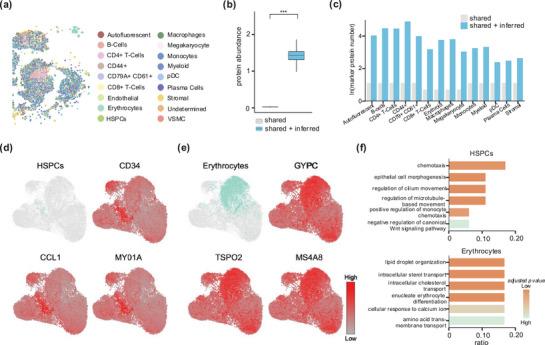
EDGES advances spatial proteomics across applications in human bone marrow. a) Manual annotation of cell types of the CODEX human bone marrow dataset. b) Boxplot shows the total protein abundance per cell in the unprocessed and EDGES‐processed data (*n* = 21145 in each boxplot). The box plot center line and the box limits represent the median value and upper and lower quartiles, respectively. Box whiskers indicate the largest and smallest values no more than 1.5 times the interquartile range from the limits. The statistical *p*‐value was determined by the Wilcoxon rank‐sum test with *** representing *p* less than 2.2e‐16. c) Barplot shows the number of marker proteins between HSPCs and other cell types identified from the unprocessed data and EDGES‐processed data. (d, e) UMAP visualizations of selected marker proteins for d) HSPCs and e) erythrocytes, respectively. f) Barplots show the functional enrichment of marker proteins specific to HSPCs (top) and erythrocytes (bottom).

EDGES provides a flexible framework for integration tasks, making it applicable to diverse scenarios. To further demonstrate its versatility, we applied EDGES to a co‐detection by indexing (CODEX) human bone marrow dataset to explore its potential in spatial proteomics.^[^ ^38^ ^]^ The original CODEX data offers high‐resolution spatial distributions of 49 proteins along with cell type annotations (**Figure** **5**a). After executing EDGES, over 2000 proteins were inferred with the average abundance levels per cell significantly improved (Figure 5b, Wilcoxon rank‐sum test *p* < 2.2e‐16).

Given that hematopoiesis is the primary function of human bone marrow, and hematopoietic stem and progenitor cells (HSPCs) possess the ability to self‐renewal and differentiate into diverse blood cell lineages,^[^ ^39^, ^40^, ^41^ ^]^ we focus our analysis on HSPCs and their derived erythrocytes. Notably, EDGES enhanced both the total number of marker proteins and the number of proteins with significantly elevated abundance, distinguishing HSPCs and erythrocytes from other cell types (Figure 5c; Figure S9, and Table S5, Supporting Information). Specifically, in the raw data, only one HSPC marker protein, CD34, was identified. In contrast, EDGES‐enhanced data revealed 19 additional marker proteins, all of which exhibited spatial distributions consistent with HSPCs (Figure 5d). Similar improvements were also observed for erythrocytes, highlighting the effectiveness of EDGES in enhancing protein detection and spatial resolution (Figure 5e; Figure S10, Supporting Information).

We next explored the functional characterization of the newly identified marker proteins. Corresponding enrichment analysis emphasized the roles of HSPC markers in regulating cell motility, metabolic processes, and cytoskeletal dynamics, all of which contribute to HSPC homing, differentiation potential, and self‐renewal capacity (Figure 5f; Table S5, Supporting Information).^[^ ^42^, ^43^, ^44^, ^45^ ^]^ Additionally, TSPO2, an erythroid lineage‐specific marker, played a critical role in erythropoiesis by regulating cholesterol transport dynamics, which is essential for erythrocyte differentiation and functional maintenance.^[^ ^46^ ^]^ Further functional analysis revealed that erythrocyte‐associated markers are involved in lipid metabolic pathways necessary for erythroid maturation and homeostasis maintenance (Figure 5f; Table S5, Supporting Information).^[^ ^47^ ^]^

### 2.6 EDGES Characterizes Novel Spatial Expression Patterns in the Mouse Primary Visual Cortex

**Figure 6:**
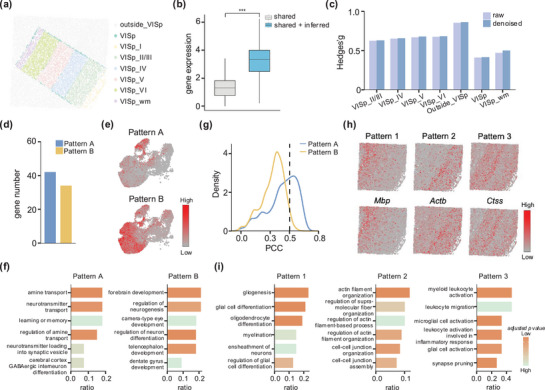
Analysis results on mouse primary visual cortex data. a) Manual annotation of distinct layers of the BaristaSeq mouse primary visual cortex dataset. b) Boxplot shows the total gene expression per cell in the unprocessed data and EDGES‐processed data (*n* = 11426 in each boxplot). The box plot center line and the box limits represent the median value and upper and lower quartiles, respectively. Box whiskers indicate the largest and smallest values no more than 1.5 times the interquartile range from the limits. The statistical *p*‐value was determined by the Wilcoxon rank‐sum test with *** representing *p* less than 2.2e‐16. c) Barplot shows the Hedges'g values of *Dcn* between VISp_I and other layers before and after EDGES denoising. d) Barplot shows the number of measured genes specific to Patterns A and B. e) UMAP visualizations of the expression Patterns A and B. f) Barplots show the functional enrichment of measured genes specific to Patterns A and B. g) Density curves display the correlation distributions of inferred gene specific to Patterns A and B. h) Visualizations of newly identified Patterns 1–3 (top) and representative genes (bottom). i) Barplots show the functional enrichment of inferred genes specific to Patterns 1, 2, and 3.

Understanding spatial gene expression patterns is essential for elucidating the functional architecture of the tissues under investigation. To improve gene coverage and uncover novel spatial gene expression patterns, we applied EDGES to a BaristaSeq^[^ ^48^ ^]^ dataset of the mouse primary visual cortex (VISp). The original dataset includes manually annotated cortical layers and spatial distributions of 80 genes (**Figure** **6**a). EDGES successfully inferred the expression of over 2000 genes and enhanced the specificity of marker genes for each layer, including VISp_I, VISp_IV, VISp_V, VISp_VI, and VISp_wm (Figure 6b,c; Figures S11 and S12, Supporting Information).

We first identified two distinct expression patterns in the original data, labeled Pattern A and Pattern B. Spatial and UMAP visualizations revealed that these patterns exhibited different spatial expression profiles (Figure 6d,e; Figure S13a, Supporting Information). Specifically, Pattern A, which included 42 genes, was predominantly clustered in VISp_V and VISp_VI, while Pattern B, consisting of 34 genes, was primarily localized in VISp. Functional enrichment analysis further indicated that genes in Pattern A were mainly associated with neural transmission, whereas genes in Pattern B were closely linked to brain development and neural regulation (Figure 6f).

We employed a correlation‐based strategy to examine the associations between the inferred genes and known expression patterns (Methods). As a result, 765 previously undetected genes were associated with Pattern A, while 65 were linked to Pattern B (Figure 6g). Beyond these associations, EDGES characterized five additional expression patterns among other inferred genes, each enriched in distinct cortical layers (Figure 6h; Figure S13b, Supporting Information). Specifically, Pattern 1 was localized in VISp_VI, Pattern 2 in VISp_I and VISp_II/III, Pattern 3 in VISp_I, VISp_II/III, and VISp_IV, Pattern 4 in VISp_V, VISp_VI, and VISp_wm, while Pattern 5 exhibited a sparse distribution.

We further conducted enrichment analyses to validate the biological relevance of the newly identified patterns in relation to layer‐specific functions. For example, Pattern 1 was enriched for oligodendrocyte differentiation and featured *Mbp*, a canonical marker gene essential for oligodendrocyte maturation and neural signal transmission.^[^ ^49^ ^]^ Pattern 2 genes were linked to cytoskeletal regulation, including *Actb*, whose product is a key component of microfilaments essential for cellular motility and intercellular connectivity (Figure 6i; Table S6, Supporting Information).^[^ ^50^ ^]^ In contrast, Pattern 3 was primarily associated with leukocyte activation and migration, exemplified by *Ctss*, a key regulator of immunomodulatory responses (Figure 6i; Table S6, Supporting Information).^[^ ^51^, ^52^ ^]^ Rather than overlapping with established neuron‐dominated patterns, these newly identified spatial architectures revealed distinct non‐neuronal cellular functions, complementing manually annotated layers with additional biological insights (Figure 6i; Figure S13c, and Table S6, Supporting Information).

## 3 Discussion

Imaging‐based ST technologies enable simultaneous profiling of gene expression and spatial localization at single‐cell resolution, but their utility is often limited by low gene detection capacity and technical noise. To address these challenges, we developed EDGES, a spatially constrained NMF framework that jointly predicts undetected gene expression and denoises measured transcriptional profiles. Comprehensive benchmarking demonstrated that EDGES consistently outperforms existing methods in both predictive performance and denoising efficacy across a range of datasets. The versatility of EDGES facilitates the identification of novel biological markers across species and spatially resolved expression patterns.

The success of EDGES is mainly attributed to the mutually coupled decomposition strategy. By jointly decomposing ST and scRNA‐seq datasets, EDGES effectively captures modality‐specific biological variation while preserving co‐expression patterns across modalities. In this framework, *X*_2_ serves as a “bridge”, linking the shared genes in the ST data with undetected genes in the reference scRNA‐seq data, thereby enabling the transfer of high‐resolution transcriptional features from scRNA‐seq data to ST data. Furthermore, the independent outputs of denoised spatial profiles and predicted gene expression allow for flexible application to a broad range of downstream analytical tasks. Another essential contributor to the superior performance of EDGES lies in its incorporation of spatial information through Laplacian regularization. This regularization preserves the underlying tissue architecture by promoting similarity among neighboring spatial cells within the low‐dimensional latent space. In contrast to KNN‐based methods that depend solely on expressions, EDGES employs a more advanced integration strategy that combines expression profiles with spatial information, leading to a more accurate reconstruction of gene expression (Figure S14, Supporting Information).

While EDGES demonstrates strong performance in gene prediction and denoising, there remain several opportunities for further enhancement. One limitation is that the current framework does not explicitly account for batch effects between ST and scRNA‐seq datasets. In cases where batch effects are pronounced, such variation may adversely impact the predictive accuracy. As a practical recommendation, users are encouraged to apply established batch correction algorithms, such as Harmony,^[^ ^53^ ^]^ during data preprocessing to mitigate potential biases and improve the model's robustness. Furthermore, although EDGES exhibits competitive computational efficiency (Table S7, Supporting Information), its computational burden increases with the number of spatial cells due to the matrix decomposition. To extend its applicability for ultra‐large ST datasets, incorporating strategies such as block‐wise parallelization or down‐sampling may be necessary. In future studies, we aim to expand the EDGES framework to integrative analyses of spatial multi‐omics datasets, including spatial ATAC‐seq^[^ ^54^ ^]^ and spatial CITE‐seq,^[^ ^55^ ^]^ thereby broadening its applicability across diverse molecular modalities. Additionally, EDGES can generate low‐dimensional representations for both spatial cells and single cells. Using the low‐dimensional representations of these cells for clustering, followed by comparison with spatial domain segmentation and single‐cell clustering algorithms, could be another promising analytical strategy.^[^ ^56^, ^57^, ^58^, ^59^, ^60^ ^]^

## 4 Experimental Section

### Data Preprocessing

In this study, the standard pipeline from Seurat^[^ ^61^ ^]^ (version 5.1.0) was followed to preprocess the scRNA‐seq data. The low‐quality cells were first removed with Seurat parameters “min.feature = 200” to exclude cells with fewer than 200 detected genes. Next, the expression matrix was normalized using the “NormalizeData” function with the “LogNormalize” method. After retaining the shared genes with the ST data, the 2000 highly expressed genes in the scRNA‐seq data were selected to reduce unnecessary noise.

For the gene expression profiles of ST data, low‐quality cells were filtered using the same quality control procedure as for scRNA‐seq data. Next, the data were normalized using the following transformation:

(1) $$D_{ij}=\;\log\left(\bar{N}\times \frac{C_{ij}}{\sum_{j=1}^{C}C_{ij}}+1\right)$$

where *C_ij_* and *D_ij_* represent the raw and normalized expressions for gene *i* in cell *j*, respectively. $\bar{N}$ is the mean number of detected transcripts per cell. This normalization accounts for differences in sequencing depth and gene expression variance. The detailed information of the original datasets used in this study was summarized in Table S1 (Supporting Information).

### Matrix Decomposition in EDGES

Denote $X_{1}\in R^{S\times c_{1}}$ as the ST data, where *S* is the number of shared genes between the ST data and reference scRNA‐seq data, and *c*_1_ is the number of cells in the ST data. Based on whether genes were present in the ST data, the reference scRNA‐seq data can be partitioned as $X_{2}\in R^{S\times c_{2}}$ and $X_{3}\in R^{U\times c_{2}}$, where *U* is the number of genes uniquely present in the reference scRNA‐seq data but absent in the ST data, and *c*_2_ is the number of cells in the scRNA‐seq data. It was hypothesized that *X*_2_ can serve as a “bridge” to connect the shared genes in the ST data with undetected genes in the reference scRNA‐seq data. This linkage ensures that the shared genes maintain identical low‐dimensional representations across modalities. Meanwhile, the single cells in scRNA‐seq data preserve consistent representations within their modality. To achieve this, EDGES employs a mutually coupled decomposition strategy based on NMF:

(2) $$X_{1}\approx W_{1}H_{1}$$

(3) $$X_{2}\approx W_{1}H_{2}$$

(4) $$X_{3}\approx W_{2}H_{2}$$

where $W_{1}\in R^{S\times d}$ and $W_{2}\in R^{U\times d}$ contain the shared and unique gene representations. $H_{1}\in R^{d\times c_{1}}$ and $H_{2}\in R^{d\times c_{2}}$ capture the cell representations from the ST data and the reference scRNA‐seq data, respectively. *d* is the dimension of the number of predefined patterns.

EDGES assumes that matrix factorization can effectively capture common expression patterns among cells. In this framework: *H*_1_ and *H*_2_ characterize the membership degree of cells to different patterns, their elements were constrained to be non‐negative values to ensure interpretability and biological relevance. As for *W*_1_ and *W*_2_, they represent the characteristic gene expression profiles of each pattern. Since the input gene expression values were all non‐negative, EDGES enforces non‐negativity on *W*_1_ and *W*_2_ as well as to maintain consistency during decomposition.

The NMF forces *W*_1_ to encode the same underlying features of the shared genes across both *X*_1_ and *X*_2_. Besides, *H*_2_ guarantees that the cellular heterogeneity representations in *X*_2_ align exactly with those in *X*_3_. The above decompositions were formulated as an optimization problem to minimize the reconstruction error:

(5) $$\min_{W_{1},W_{2},H_{1},H_{2}\geq 0}\theta_{1}∥X_{1}-W_{1}H_{1}{∥}_{F}^{2}+∥X_{2}-W_{1}H_{2}{∥}_{F}^{2}+\theta_{2}{∥X_{3}-W_{2}H_{2}∥}_{F}^{2}$$

where θ_1_ and θ_2_ are model hyperparameters used to balance the decompositions, and ‖ · ‖_*F*_ is the Frobenius norm.

### Spatial and Sparse Regularization

To effectively incorporate the spatial information of cells in the ST data, a cell–cell proximity graph *G* was constructed based on the mutual *k* nearest neighbors, determined by the Euclidean distance between the spatial coordinates of cells. To preserve the manifold structure of *G* and ensure that proximal cells have similar low‐dimensional representations, a spatial regularization term based on the graph Laplacian was introduced:

(6) $$tr\left(H_{1}L_{1}H_{1}^{T}\right)$$

where *L*_1_ = *I* − *D* ^−(1/2)^ *AD* ^−(1/2)^ is the normalized Laplacian matrix. *A* was the adjacency matrix of *G*. *I* was the identity matrix, and *D* was a diagonal matrix where each diagonal entry represents the sum of the corresponding row in *A*.

Since cells typically belong to only one or a few specific patterns (such as cell types or functional regions) rather than being a mixture of many, the following sparse regularization term constraints were imposed on *H*_1_ and *H*_2_ to ensure that each cell was associated with only a limited number of patterns. This approach can also reflect cellular heterogeneity, preserves the specificity of different patterns, and effectively avoids pattern ambiguity caused by overfitting:

(7)

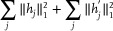

where *h_j_* and $h_{j}^{\prime }$ are the *j*‐th columns of *H*_1_ and *H*_2_, respectively, and ‖ · ‖_1_ represents the *L*_1_‐norm.

Due to the multifunctional nature of genes (such as producing different types of molecules, participating in complex regulatory networks, and influencing diverse phenotypes), genes were often involved in multiple patterns rather than being restricted to a single one. Therefore, sparsity constraints were not imposed on *W*_1_ and *W*_2_, allowing the matrices to retain their original form in order to better preserve the combinatorial characteristics of genes across various patterns.

### EDGES Formulation and Optimization

The overall objective function of EDGES consists of three key components: matrix decompositions, spatial regularization, and sparse regularization. The final model of EDGES can be formulated as a constrained multi‐objective optimization problem:

(8) $$\begin{array}{cc} \min_{W_{1},W_{2},H_{1},H_{2}\geq 0}F\left(W_{1},W_{2},H_{1},H_{2}\right) & =\theta_{1}∥X_{1}-W_{1}H_{1}{∥}_{F}^{2}+{∥X_{2}-W_{1}H_{2}∥}_{F}^{2}\\ & \;+\theta_{2}{∥X_{3}-W_{2}H_{2}∥}_{F}^{2}+ \end{array}$$

(9)

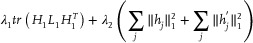

where λ_1_ and λ_2_ are model hyperparameters used to control the strength of spatial and sparse regularization, respectively.

Since the above objective function was not convex, it was unrealistic to expect a standard optimization algorithm to find the global minimum. Therefore, the classical multiplicative updating algorithm developed for NMF was extended to effectively explore the local minimum of the above optimization problem. The algorithmic framework for EDGES was outlined below, and the detailed mathematical derivations were summarized in Note S2 (Supporting Information).

Algorithm 1 **Algorithmic Framework for EDGES**

**Table:** 

Input: ST data and reference scRNA‐seq data
Step1: According to the shared genes between the ST data and reference scRNA‐seq data, partition the inputs matrices into $X_{1}\in R^{S\times c_{1}}$, $X_{2}\in R^{S\times c_{2}}$, and $X_{3}\in R^{U\times c_{2}}$. Construct the normalized Laplacian matrix *L*_1_ based on spatial coordinates.
Step2: Initialize $W_{1}\in R^{S\times d}$, $W_{2}\in R^{U\times d}$, $H_{1}\in R^{d\times c_{1}}$ and $H_{2}\in R^{d\times c_{2}}$ with non‐negative values, hyperparameters θ_1_, θ_2_, λ_1_, λ_2_ and set the iteration index *t* = 0 and convergence threshold τ = 10^−7^.
Step3: Fix *H*_1_ and *H*_2_, update *W*_1_ and *W*_2_ with $w_{ij}^{1}\leftarrow w_{ij}^{1}\frac{{\left[\theta_{1}X_{1}H_{1}^{T}+X_{2}H_{2}^{T}\right]}_{ij}}{{\left[\theta_{1}W_{1}H_{1}H_{1}^{T}+W_{1}H_{2}H_{2}^{T}\right]}_{ij}},$ $w_{ij}^{2}\leftarrow w_{ij}^{2}\frac{{\left[X_{3}H_{2}^{T}\right]}_{ij}}{{\left[W_{2}H_{2}H_{2}^{T}\right]}_{ij}}.$
Step4: Fix *W*_1_ and *W*_2_, update *H*_1_ and *H*_2_ with $h_{ij}^{1}\leftarrow h_{ij}^{1}\frac{{\left[\theta_{1}W_{1}^{T}X_{1}\right]}_{ij}}{{\left[\theta_{1}W_{1}^{T}W_{1}H_{1}+\lambda_{2}e_{d\times d}H_{1}+\lambda_{1}H_{1}L_{1}\right]}_{ij}},$ $h_{ij}^{2}\leftarrow h_{ij}^{2}\frac{{\left[W_{1}^{T}X_{2}+\theta_{2}W_{2}^{T}X_{3}\right]}_{ij}}{{\left[W_{1}^{T}W_{1}H_{2}+\theta_{2}W_{2}^{T}W_{2}H_{2}+\lambda_{2}e_{d\times d}H_{2}\right]}_{ij}},$ where *e*_*d* × *d*_ is a matrix with all elements equal to 1.
Step5: Repeat Steps 2–3 until the following stopping criteria are satisfied: $\frac{F\left(W_{1}^{t},W_{2}^{t},H_{1}^{t},H_{2}^{t}\right)-F\left(W_{1}^{t+1},W_{2}^{t+1},H_{1}^{t+1},H_{2}^{t+1}\right)}{F\left(W_{1}^{1},W_{2}^{1},H_{1}^{1},H_{2}^{1}\right)-F\left(W_{1}^{t+1},W_{2}^{t+1},H_{1}^{t+1},H_{2}^{t+1}\right)}\leq \tau .$
Output: Factorized matrices ${\hat{W}}^{\left(1\right)}$, ${\hat{W}}^{\left(2\right)}$, ${\hat{H}}^{\left(1\right)}$, ${\hat{H}}^{\left(2\right)}$.

After obtaining the factorized matrices ${\hat{W}}^{\left(1\right)}$, ${\hat{W}}^{\left(2\right)}$, ${\hat{H}}^{\left(1\right)}$, ${\hat{H}}^{\left(2\right)}$, EDGES can predict the expressions of undetected genes in the ST data by ${\hat{W}}^{\left(2\right)}\cdot {\hat{H}}^{\left(1\right)}$, and denoise the expressions of the measured genes by ${\hat{W}}^{\left(1\right)}\cdot {\hat{H}}^{\left(1\right)}$.

### Implementation of EDGES

In real applications, the latent dimension was set to *d*  =  20 and mutual nearest neighbors *k*  =  5. The latent dimension *d* was determined based on sensitivity analysis, which identified *d*  =  20 as the optimal value that achieved the best predictive performance of EDGES (Figure S15, Supporting Information). The matrices *W*_1_, *W*_2_, *H*_1_ and *H*_2_ were initialized with non‐negative values sampled uniformly from the interval [0, 1]. For the hyperparameters, the optimal values were determined through grid search, guided by prediction performance on cross‐validation experiments (Figure S16, Supporting Information), and the default values of the hyperparameters were set as: θ_1_ = 10^−1^, θ_2_ = 10^−4^, λ_1_ = 10^−5^, λ_2_ = 10. EDGES was implemented using MATLAB (version 2020a).

### Conduction of Cross‐Validation Experiments

In this study, *K*‐fold cross‐validation was performed to evaluate the predictive performance of measured genes for each method. Specifically, *K* − 1 folds were used for training and the remaining fold for validation, iterating this process *K* times to obtain predicted expressions for all measured genes. In practical applications, *K* was chosen based on the number of measured genes: *K*  =  3 for ST datasets with fewer than 50 genes, *K*  =  5 for ST datasets with 50 to 100 genes, and *K*  =  10 for all other cases. The gene‐level predictive performance was evaluated by calculating the similarity between the predicted and measured expressions for each gene.

The cell‐level predictive performance was assessed using the following strategy: After obtaining the predicted expressions for a specific fold during cross‐validation, a full gene expression vector for each cell was constructed by concatenating the predicted expressions from that fold with the original expressions of genes from the remaining folds. The similarity between this reconstructed vector and the measured expression vector of the cell was then computed. This strategy avoids potential variations in scale or distribution across folds and more accurately captures a method's ability to preserve the cell's original expression pattern when only a subset of genes was substituted.

### Evaluation Metrics

The predictive performance of each method was evaluated using the Pearson Correlation Coefficient (PCC), Structural similarity index (SSIM), Root Mean Square Error (RMSE), and Jensen‐Shannon divergence (JS). For these metrics, higher PCC and SSIM values, along with lower RMSE and JS values, indicate better agreement between predicted and measured gene expression. Besides, the evaluation pipeline from^[^ ^10^ ^]^ was followed to compute an aggregated AS based on PCC, SSIM, RMSE, and JS. Specifically, for each benchmarking dataset, the average PCC, SSIM, RMSE, and JS across all measured genes predicted by each method were first calculated. Then, the PCC and SSIM values in ascending order to obtain *RANK_PCC_* and *RANK_SSIM_*, while RMSE and JS values were ranked in descending order to get *RANK_RMSE_* and *RANK_JS_*. Finally, the AS for each method was defined as:

(10) $$AS=\frac{1}{4N}\left(RANK_{PCC}+RANK_{SSIM}+RANK_{RMSE}+RANK_{JS}\right)$$

where *N* is the number of methods. This aggregated AS ranges from $\frac{1}{N}$ to 1, with a higher score indicating better predictive performance. The Homo, NMI, AMI, and ARI were employed to quantify the clustering performance of each method based on the predicted gene expression profiles. These metrics assess the alignment between predicted clusters and ground‐truth labels, with higher values indicating better performance. Moran's *I* statistic was used to evaluate different denoising approaches by measuring the spatial autocorrelation of the identified spatially variable genes. Moran's *I* ranges from ‐1 to 1, where a higher value indicates stronger spatial clustering, while lower values suggest a more random or dispersed distribution.^[^ ^62^ ^]^ Hedges'g, a bias‐corrected effect size metric that quantifies the standardized difference between two independent groups with unequal sample sizes, was incorporated, where a higher value indicates a greater magnitude of difference. The detailed computational formulas for all evaluation metrics were provided in Note S3 (Supporting Information).

### Competing Methods

In this study, the predictive performance of EDGES was benchmarked against four state‐of‐the‐art methods, including Tangram, SpaGE, stPlus, and LIGER. Besides, three methods (EAGS, SPCS, and Sprod) designed for spatially resolved transcriptomics were selected to evaluate the denoising of the measured gene expression profiles. More details on the methods and configurations were summarized in Note S4 and Table S2 (Supporting Information).

### Identification of the Differentially Expressed Genes

Differentially expressed genes (DEGs) for specific cell types were identified using the edgeR^[^ ^22^ ^]^ R package (version 4.2.1). The generalized linear model likelihood ratio test was performed using the “glmLRT” function, and multiple testing correction was applied using the Benjamini‐Hochberg (BH) method (adjust.method = “BH”). Finally, genes with |log_2_ Fold Change| > 1 and false discovery rate < 0.05 were considered as DEGs.

### Clustering Analysis

Cluster analysis was conducted on both genes and cells following the standard Seurat pipeline. The number of principal components was set to 10 in the “RunPCA” function, and the resolution was set to 0.5 in the “FindClusters” function. Clusters were identified using Louvain clustering with a fixed random seed.

Figure 2g was based on genes with measured spatial expressions and performed clustering on the predicted expressions obtained through cross‐validation. The clustering results derived from the measured spatial expressions of osmFISH cells were used as clustering labels (serving as the gold standard), allowing the computation of clustering metrics. This provides an alternative perspective to evaluate the similarity between the predicted and measured spatial expressions.

As for Figure 3f, the clustering metrics were calculated by comparing the true cell type labels from osmFISH with the clustering results obtained using either “shared” genes or “shared + inferred” genes. The goal of Figure 3f was to evaluate which method better captures the intrinsic cellular heterogeneity of the data after incorporating the inferred gene expressions, which were entirely unobserved.

### Pathway Enrichment Analysis

The Gene Ontology analysis was conducted by the “enrichGO” function in the ClusterProfiler^[^ ^63^ ^]^ R package (version 4.12.6), focusing on biological processes. The terms with adjusted *p*‐values less than 0.05 based on the BH correction were considered enriched pathways.

### Calculation of the Gene Abundance Scores

The gene abundance score was defined by summing the low‐dimensional representations of each gene obtained from ${\hat{W}}^{\left(1\right)}$. This score integrates the effective information from the original gene expression profile, enabling the comprehensive assessment of gene importance and the selection of essential genes.

### Identification of the Spatially Variable Genes

Spatially variable genes (SVGs) were identified from each denoised dataset using Hotspot^[^ ^32^ ^]^ and SPARK,^[^ ^37^ ^]^ respectively. The “hotspot.Hotspot” function (model = “none”) compiled in the hotspot Python package (version 0.9.0) was used to create a Hotspot object. Then the standard analysis pipeline with default parameters was then followed to report the top 15 SVGs with the lowest *p* values for each denoising method. For SPARK analysis, the “spark.vc” and “spark.test” functions in the SPARK R package (version 1.1.1) was used with the default parameters to identify the SVGs. Similarly, only the top 15 SVGs with the lowest *p*‐values were considered for each denoising method.

### Identification of Gene Expression Patterns

The expression Patterns A and B were obtained based on the gene clusters following the standard Seurat pipeline. For an undetected gene, the PCC was assessed between its predicted expression and the average expression of Pattern A or Pattern B, respectively. A gene was assigned to a known expression pattern if its PCC exceeded 0.5 and was assigned to the pattern with the highest PCC if it could be assigned to multiple patterns. Novel gene expression patterns were identified by clustering the genes that could not be assigned to known expression patterns, with each cluster representing a distinct novel gene expression pattern.

### Statistical Analysis

The statistical *p*‐values reported in Figure 2d were calculated using the Student’*s t*‐test. For pathway enrichment analyses, *p*‐values were computed using Fisher's exact test and adjusted using the BH procedure. All other *p*‐values mentioned in the text were calculated using the two‐sided Wilcoxon rank‐sum test, implemented with the “wilcox.test” function in R using default parameters.

## Conflict of Interest

The authors declare that they have no competing interests

## Author Contributions

D.S. and L.Y.W. conceived the idea and supervised the study. J.Z. implemented the algorithm. J.Z. and Y.C. performed the analyses. J.Z., D.S., and L.Y.W. interpreted the results. J.Y. and F.Y. provided scientific insights on the applications. J.Z., J.Y., and D.S. wrote the manuscript with feedback from all other authors. All authors read and approved the final manuscript.

## Supporting information



Supporting Information

Supporting Tables

## Data Availability

All datasets analyzed in this study are publicly available. The corresponding descriptions and pre‐processing steps can be found in Note S1 (Supporting Information). The open‐source MATLAB and Python codes for EDGES are available at GitHub: https://github.com/SDU‐Math‐SunLab/EDGES.
