# Supplementary material for: Enhancing Spatial Transcriptomics via Spatially Constrained Matrix Decomposition with EDGES
Source: Adv Sci (Weinh). 2025 Aug 21;12(42):e08346. doi: 10.1002/advs.202508346 (PMC12622516; doi:10.1002/advs.202508346)
Supplement: Supplementary file 1 — Supporting Information [file ADVS-12-e08346-s002.pdf]

Supporting Information

Enhancing Spatial Transcriptomics via Spatially Constrained Matrix Decomposition with EDGES

Jinyue Zhao[1], Jiating Yu[2], Yuqing Cao[1], Fan Yuan[3], Ling-Yun Wu[4, 5, *] and Duanchen Sun[1, 6, *]

1 School of Mathematics, Shandong University, Jinan 250100, China;

2 School of Mathematics and Statistics, Nanjing University of Information Science & Technology, Nanjing 210044, China;

3 School of Mathematics and Information Science, Yantai University, Yantai 264005, China;

4 State Key Laboratory of Mathematical Sciences, Academy of Mathematics and Systems Science, Chinese Academy of Sciences, Beijing 100190, China;

5 School of Mathematical Sciences, University of Chinese Academy of Sciences, Beijing 100049, China;

6 Shandong Key Laboratory of Cancer Digital Medicine, Jinan 250033, China;

* These authors jointly supervised this work: Ling-Yun Wu, Duanchen Sun. Email: lywu@amss.ac.cn, dcsun@sdu.edu.cn.

Contents

1. Supplementary Note 1 3

2. Supplementary Note 2 7

3. Supplementary Note 3 9

4. Supplementary Note 4 12

5. Supporting results for main Figure 2 13

6. Supporting results for main Figure 3 16

7. Supporting results for main Figure 4 18

8. Supporting results for main Figure 5 20

9. Supporting results for main Figure 6 22

10. Comparison between EDGES with a KNN-based approach 25

11. Computational efficiency 26

12. Selection of the latent dimension d in EDGES 27

13. Hyper-parameter selection of EDGES……………………………………………………28

1. Supplementary Note 1

1.1 Spatial transcriptomics & proteomics data

osmFISH data. The osmFISH dataset used in this study was retrieved from https://linnarssonlab.org/osmFISH/. The expressions and cell locations were extracted from .loom source files. This data was sequenced from the somatosensory cortex of mice and contains 33 genes and 6,471 cells, which collectively span all anatomical regions within the tissue.

MERFISH data (mouse). The MERFISH dataset was downloaded from https://github.com/spacetx-spacejam/data/. This dataset was obtained from the mouse primary visual cortex (VISp), capturing expression profiles of 268 genes across 2,399 cells, with a transcriptional sparsity of 0.75.

Exseq data. The Exseq dataset was downloaded from https://github.com/spacetx-spacejam/data. This dataset derived from the same tissue specimens as the MERFISH dataset, comprises spatially resolved expression profiles of 42 genes across 1,154 cells, exhibiting a transcriptional sparsity of 0.4.

seqFISH data (gastrulation). The seqFISH dataset was downloaded from https://content.cruk.cam.ac.uk/jmlab/SpatialMouseAtlas2020/. This dataset was collected during the gastrulation stage of mice development and comprises expression profiles of 351 genes across 57,536 cells. It includes experimentally validated cell type labels and annotated cellular subpopulations. We utilized data from slice 2 (z = 2), which comprises 31,578 cells.

STARmap data. The STARmap dataset was downloaded from https://www.starmapresources.com/data. This dataset, derived from the mouse visual cortex, contains 1,020 genes and 1,549 cells, with a sparsity of 0.79.

Xenium data (breast cancer). The human breast cancer Xenium dataset was downloaded from 10x Genomics (https://www.10xgenomics.com/products/xenium-in-situ/preview-dataset-human-breast): Xenium In Situ Sample 1, Replicate 1. This dataset contains 313 genes and 167,780 cells. For analysis, we selected a subset of cells based on their spatial coordinates (x < 200 and y < 700).

seqFISH data (embryon). The seqFISH dataset was downloaded from https://zenodo.org/record/3735329#.YY69HZMza3J. This dataset was derived from mouse embryonic tissue and includes 45 genes and 175 cells, with a sparsity of 0.17.

seqFISH data (hippocampus). The seqFISH dataset was downloaded from https://ars.els-cdn.com/content/image/1-s2.0-S0896627316307024-mmc6.xlsx. This dataset was derived from the mouse hippocampus and contains 249 genes and 3,585 cells, with a sparsity of 0.06.

seqFISH+ data. The seqFISH+ dataset was downloaded from https://github.com/CaiGroup/seqFISH-PLUS. This dataset was obtained from the mouse

cortex and includes 10,000 genes and 524 cells, with a sparsity of 0.67.

MERFISH data (human). The MERFISH dataset was downloaded from https://www.pnas.org/doi/suppl/10.1073/pnas.1912459116/suppl_file/pnas.1912459116.sd12.csv. This dataset was derived from a human osteosarcoma sample and contains 12,903 genes and 645 cells, with a sparsity of 0.39.

Xenium data (melanoma). The Xenium dataset was downloaded from the Gene Expression Omnibus (GEO) with accession number GSE286964. This dataset was obtained from the human melanoma with 541 genes and 148,741 cells. For analysis, we selected a subset of cells based on their spatial coordinates (500 < x < 3,000 and 500 < y < 3,000).

CODEX data. The CODEX human bone marrow dataset was downloaded from https://plus.figshare.com/collections/_/7174914. We used the H33 sample that contains proteomic profiles of 49 proteins across 21,145 cells, with paired single-cell RNA sequencing (scRNA-seq) data generated from the same tissue specimen.

BaristaSeq data. The BaristaSeq dataset was downloaded from https://github.com/spacetx-spacejam/data. This dataset was obtained from the mouse VISp with 80 genes and 11,426 cells.

1.2 Reference scRNA-seq data

Zeisel's data. Zeisel's scRNA-seq data was collected using the same source as the osmFISH dataset, available at https://linnarssonlab.org/cortex/. This dataset, generated from the superficial dorsal horn of the murine spinal cord, comprises transcriptional profiles of 19,972 genes across 3,005 cells, exhibiting a transcriptional sparsity of 0.81.

AllenVISp data. AllenVISp's scRNA-seq dataset, generated from the VISp of adult mice, profiles 15,414 cells and 45,768 genes. This data was retrieved from the Allen Brain Map portal (https://portal.brain-map.org/atlases-and-data/rnaseq/mouse-v1-and-alm-smart-seq) with a transcriptional sparsity of 0.81.

MouseGastrulation data. The released version of mouse gastrulation scRNA-seq data is obtained from the "MouseGastrulationData" R package (version 1.22.0) from the Bioconductor. This data contains 19,103 genes and 4,651 cells.

Breast cancer data. The breast cancer scRNA-seq dataset was downloaded from the GEO under accession number GSE278793. It was derived from human breast tissue and contains 18,082 genes and 14,096 cells.

Mouse cell atlas. The mouse cell atlas (MCA) scRNA-seq data was obtained from https://figshare.com/articles/dataset/MCA_DGE_Data/5435866. It contains 16,477 genes and 9,991 cells, with a sparsity of 0.96.

Hippocampus data. The hippocampus and prefrontal cortex scRNA-seq dataset was downloaded from the GEO under accession number GSE158450. This dataset was obtained from the mouse hippocampus and contains 16,384 genes and 8,596 cells, with

a sparsity of 0.90.

Osteosarcoma tissue data. The osteosarcoma tissue scRNA-seq dataset was obtained from the GEO under accession number GSE152048. This dataset was derived from human osteosarcoma and contains 19,098 genes and 9,234 cells, with a sparsity of 0.87.

Melanoma data. The melanoma scRNA-seq data was obtained from the GEO under accession number GSE291160. This dataset was derived from human melanoma and contains 19,936 genes and 2,000 cells.

GSE253355. This scRNA-seq data was derived from the GEO with accession number GSE253355. This data was analyzed as a paired dataset with CODEX data and consists of 33,538 genes and 8,661 cells.

Table S1. Detailed information of datasets used in this study.

Spatial/Protein and scRNA-seq dataset pair	Spatial/Protein dataset		scRNA-seq dataset	
	#cells	#genes/proteins	#cells	#genes
osmFISH_Zeisel (osmFISH_Z)	6,471	33	3,005	19,972
osmFISH_AllenVISp (osmFISH_A)	6,471	33	15,414	45,768
MERFISH_AllenVISp (MERFISH)	2,399	268	15,414	45,768
Exseq_AllenVISp (Exseq)	1,154	42	15,414	45,768
seqFISH_MouseGastrulation (seqFISH)	57,536	351	4,651	19,103
STARmap_AllenVISp (STARmap)	1,549	1,020	15,414	45,768
Xenium_GSE278793 (Xenium_B)	167,780	313	14,096	18,082
seqFISH_Mouse_cell_atlas (seqFISH_MCA)	175	45	9,991	16,477
seqFISH_GSE158450 (seqFISH_H)	3,585	249	8,596	16,384
seqFISH+_AllenVISp (seqFISH+)	524	10,000	15,414	45,768
MERFISH_GSE152048 (MERFISH_O)	645	12,903	9,234	19,098
Xenium_GSE291160 (Xenium_M)	148,741	541	2,000	19,936
CODEX_GSE253355	21,145	49	8,661	33,538
BaristaSeq_AllenVISp	11,426	80	15,414	45,768

*The abbreviations in parentheses indicate the dataset identifiers used in this study.

2. Supplementary Note 2

In this section, we provided the detailed mathematical derivations of EDGES.

Consider the constrained multi-objective optimization problem:

$$\min_{W_1,W_2,H_1,H_2\geq 0} F(W_1,W_2,H_1,H_2) = \theta_1\|X_1 - W_1H_1\|_F^2 + \|X_2 - W_1H_2\|_F^2 + \theta_2\|X_3 - W_2H_2\|_F^2 +$$

$$\lambda_1 tr(H_1L_1H_1^T) + \lambda_2(\sum_j \|h_j\|_1^2 + \sum_j \|h_j'\|_1^2).$$

We reformulated the objective function F based on the knowledge of linear algebra:

$$F = \theta_1 tr(X_1X_1^T) - 2\theta_1 tr(X_1H_1^TW_1^T) + \theta_1 tr(W_1H_1H_1^TW_1^T) + tr(X_2X_2^T) - 2tr(X_2H_2^TW_1^T)$$
$$+ tr(W_1H_2H_2^TW_1^T) + \theta_2 tr(X_3X_3^T) - 2\theta_2 tr(X_3H_2^TW_2^T) + \theta_2 tr(W_2H_2H_2^TW_2^T)$$
$$+ \lambda_1 tr(H_1L_1H_1^T) + \lambda_2(e_{1\times d}H_1H_1^Te_{1\times d}^T + e_{1\times d}H_2H_2^Te_{1\times d}^T),$$

where $e_{1\times d}$ is a d-dimensional row vector with all elements equal to 1. Let $(\Psi_I)_{ij}$ and $(\Phi_I)_{ij}$ be the Lagrange multipliers for the constraints $(W_I)_{ij} \geq 0$, $(H_I)_{ij} \geq 0$, $I = 1, 2$, respectively. The Lagrange L is defined as:

$$L = F + tr(\Psi_1W_1^T) + tr(\Psi_2W_2^T) + tr(\Phi_1H_1^T) + tr(\Phi_2H_2^T).$$

The partial derivatives of L with respect to W_1, W_2, H_1, H_2 are:

$$\frac{\partial L}{\partial W_1} = -2\theta_1X_1H_1^T + 2\theta_1W_1H_1H_1^T - 2X_2H_2^T + 2W_1H_2H_2^T + \Psi_1,$$

$$\frac{\partial L}{\partial W_2} = -2\theta_2X_3H_2^T + 2\theta_2W_2H_2H_2^T + \Psi_2,$$

$$\frac{\partial L}{\partial H_1} = -2\theta_1W_1^TX_1 + 2\theta_1W_1^TW_1H_1 + 2\lambda_1H_1L_1 + 2\lambda_2e_{d\times d}H_1 + \Phi_1,$$

$$\frac{\partial L}{\partial H_2} = -2W_1^TX_2 + 2W_1^TW_1H_2 - 2\theta_2W_2^TX_3 + 2\theta_2W_2^TW_2H_2 + 2\lambda_2e_{d\times d}H_2 + \Phi_2.$$

Based on the Karush-Kuhn-Tucker conditions $(\Psi_1)_{ij}(W_1)_{ij} = 0$, $(\Psi_2)_{ij}(W_2)_{ij} = 0$, $(\Phi_1)_{ij}(H_1)_{ij} = 0$ and $(\Phi_2)_{ij}(H_2)_{ij} = 0$, we get the following equations for $(W_1)_{ij}$, $(W_2)_{ij}$, $(H_1)_{ij}$ and $(H_2)_{ij}$:

$$[\theta_1W_1H_1H_1^T + W_1H_2H_2^T]_{ij}(W_1)_{ij} + [-\theta_1X_1H_1^T - X_2H_2^T]_{ij}(W_1)_{ij} = 0,$$

$$[\theta_2W_2H_2H_2^T]_{ij}(W_2)_{ij} + [-\theta_2X_3H_2^T]_{ij}(W_2)_{ij} = 0,$$

$$[\theta_1W_1^TW_1H_1 + \lambda_2e_{d\times d}H_1 + \lambda_1H_1L_1]_{ij}(H_1)_{ij} + [-\theta_1W_1^TX_1]_{ij}(H_1)_{ij} = 0,$$

$$[W_1^TW_1H_2 + \theta_2W_2^TW_2H_2 + \lambda_2e_{d\times d}H_2]_{ij}(H_2)_{ij} + [-W_1^TX_2 - \theta_2W_2^TX_3]_{ij}(H_2)_{ij} = 0,$$

where $e_{d\times d}$ is a matrix with all elements equal to 1. Then we can get the following

updating rules:

$$w_{ij}^{1} \leftarrow w_{ij}^{1}\frac{[\theta_1 X_1 H_1^T + X_2 H_2^T]_{ij}}{[\theta_1 W_1 H_1 H_1^T + W_1 H_2 H_2^T]_{ij}},$$

$$w_{ij}^{2} \leftarrow w_{ij}^{2}\frac{[X_3 H_2^T]_{ij}}{[W_2 H_2 H_2^T]_{ij}},$$

$$h_{ij}^{1} \leftarrow h_{ij}^{1}\frac{[\theta_1 W_1^T X_1]_{ij}}{[\theta_1 W_1^T W_1 H_1 + \lambda_2 e_{d\times d} H_1 + \lambda_1 H_1 L_1]_{ij}},$$

$$h_{ij}^{2} \leftarrow h_{ij}^{2}\frac{[W_1^T X_2 + \theta_2 W_2^T X_3]_{ij}}{[W_1^T W_1 H_2 + \theta_2 W_2^T W_2 H_2 + \lambda_2 e_{d\times d} H_2]_{ij}}.$$

3. Supplementary Note 3

In this section, we described the evaluation metrics used in our study.

Predictive performance metrics:

Pearson Correlation Coefficient (PCC). PCC quantifies the linear relationship between the predicted expression values and the measured expression values. It was calculated using the following equation:

$$PCC(X,Y) = \frac{\sum_{i=1}^{n}(x_i - \bar{x})(y_i - \bar{y})}{\sqrt{\sum_{i=1}^{n}(x_i - \bar{x})^2}\sqrt{\sum_{i=1}^{n}(y_i - \bar{y})^2}},$$

where $X = (x_1, x_2, \cdots, x_n)$ and $Y = (y_1, y_2, \cdots, y_n)$ represent the predicted expression values and the measured expression values, respectively. $\bar{x}$ and $\bar{y}$ stand for the mean of the corresponding values.

Structural similarity index (SSIM). The SSIM value was calculated using the following equation:

$$SSIM(X,Y) = \left(\frac{2\mu_X\mu_Y + C_1^2}{\mu_X^2 + \mu_Y^2 + C_1^2}\right) \cdot \left(\frac{2\sigma_{XY} + C_2^2}{\sigma_X^2 + \sigma_Y^2 + C_2^2}\right),$$

where X and Y are defined as above. $\mu_{\cdot}$ and $\sigma_{\cdot}^2$ represent the mean and the variance of the corresponding values. σ_{XY} is the covariance between X and Y, C_1 and C_2 are 0.01 and 0.03, respectively.

Root Mean Square Error (RMSE). RMSE measures the difference between the predicted and measured expression values. It is defined as:

$$RMSE = \sqrt{\frac{1}{n}\sum_{i=1}^{n}(x_i - y_i)^2},$$

where X and Y are defined as above.

Jensen-Shannon divergence (JS). JS uses relative information entropy to determine the difference between two distributions. The JS value was calculated using the following equation:

$$JS = \frac{1}{2}KL\left(P_X \middle\| \frac{P_X + Q_Y}{2}\right) + \frac{1}{2}KL\left(Q_Y \middle\| \frac{P_X + Q_Y}{2}\right),$$

where $P_X = [X_1, X_2, \cdots, X_n]$ and $Q_Y = [Y_1, Y_2, \cdots, Y_n]$ represent the spatial probability distribution of the predicted and measured expression values. $KL(\cdot \| \cdot)$ is the Kullback-Leibler divergence between the two probability distributions.

Clustering metrics:

Homogeneity (Homo). The Homo score assesses class purity by calculating the conditional entropy of true labels given the clustering assignments. It was calculated using the following equation:

$$Homo = 1 - \frac{H(C|K)}{H(C)},$$

where $H(C)$ is the entropy of the ground truth class labels. $H(C|K)$ denotes the conditional entropy of class labels given the clustering assignments. A higher homogeneity score (closer to 1) indicates that clusters are pure with respect to the true labels.

Normalized Mutual Information (NMI). NMI is used to evaluate the similarity between two clustering results while accounting for randomness. It was defined as:

$$NMI = \frac{2 \cdot MI(C;K)}{H(C) + H(K)},$$

where C represents the ground truth class labels. K represents the predicted clustering labels. $H(\cdot)$ denotes information entropy, and $MI(C;K)$ denotes the mutual information, measuring the amount of information shared between C and K.

Adjusted Mutual Information (AMI). AMI extends NMI with adjustments for cluster size imbalance. It was calculated by:

$$AMI = \frac{MI(C;K) - E[MI(C;K)]}{max[H(C), H(K)] - E[MI(C;K)]},$$

where the notations are the same as NMI. $E[MI(C;K)]$ denotes the expected mutual information under a random clustering assignment.

Adjusted Rand Index (ARI). ARI measures the similarity between two clustering assignments while adjusting for random chance. It was calculated using the following equation:

$$ARI = \frac{\sum_{ij}\binom{n_{ij}}{2} - \left[\sum_i \binom{a_i}{2} \sum_j \binom{b_j}{2}\right] / \binom{N}{2}}{\frac{1}{2}\left[\sum_i \binom{a_i}{2} + \sum_j \binom{b_j}{2}\right] - \left[\sum_i \binom{a_i}{2} \sum_j \binom{b_j}{2}\right] / \binom{N}{2}},$$

where n_{ij} is the number of samples in the intersection of ground truth class C_i and predicted clustering K_j. $a_i = \sum_j n_{ij}$ and $b_i = \sum_i n_{ij}$ are the sums over rows and columns of the contingency table, respectively. N is the total number of samples.

Spatially autocorrelated metric:

Moran's *I*. The Moran's *I* value was calculated using the following equation:

$$\text{Moran's } I = \frac{n}{\sum_{i=1}^{n}\sum_{j=1}^{n} w_{ij}} \cdot \frac{\sum_{i=1}^{n}\sum_{j=1}^{n} w_{ij}\,(y_i - \bar{y})}{\sum_{j=1}^{n}(y_i - \bar{y})^2},$$

where n represents the sample size, w_{ij} is an element in the spatial weight matrix W, indicating the spatial similarity between cell i and cell j. Matrix W was formed by combining the adjacency matrix constructed same as the main text and diagonal elements computed as the sum of each row of the adjacency matrix. In the weight matrix, the weights were equivalent to the distance values. y is the gene vector and $\bar{y}$ is the mean of the gene vector.

Effect size metric:

Hedges'g. The Hedges'g value was calculated using the following equation:

$$\text{Hedges'g} = \left(1 - \frac{3}{4(n_1 + n_2 - 2) - 1}\right) \cdot \frac{\bar{X}_1 - \bar{X}_2}{s_p},$$

$$s_p = \sqrt{\frac{(n_1 - 1)s_1^2 + (n_2 - 1)s_2^2}{n_1 + n_2 - 2}},$$

where $\bar{X}_1$ represents the mean of the experimental group, $\bar{X}_2$ represents the mean of the control group, s_1 and s_2 are the standard deviation of the experimental and control groups, n_1 and n_2 are the number of cells in the experimental and control groups. s_p denotes the pooled standard deviation of the two groups.

4. Supplementary Note 4

In this study, we benchmarked the predictive performance of EDGES against four state-of-the-art methods, including Tangram, SpaGE, stPlus, and LIGER. Besides, three methods (EAGS, SPCS, and Sprod) designed for spatially resolved transcriptomics were selected to evaluate the denoising of the measured gene expression profiles. We executed each method following the corresponding tutorial with the default parameters, except for SpaGE (n_pv = 11) and Sprod (lambda = 10). The details of the competing methods are summarized in Table S2.

Table S2. Details of the competing methods used in this study.

Methods	Version	Github (https://github.com/)
Tangram	1.0.4	broadinstitute/Tangram
SpaGE	1.0	tabdelaal/SpaGE
stPlus	0.0.6	xy-chen16/stPlus
LIGER	1.0.1	welch-lab/liger
EAGS	1.0.5	STOmics/EAGS
SPCS	-	Usos/SPCS
Sprod	1.0	yunguan-wang/SPROD

5. Supporting results for main Figure 2

This section provides supporting materials for the analysis of EDGES in predicting undetected genes, serving as a supplement to Figure 2 in the main text.

Figure S1 presents the Accuracy Score (AS) comparisons of EDGES against state-of-the-art computational methods across different datasets, demonstrating EDGES' superior predictive performance.

Figure S2 shows the predictive performance comparisons of EDGES and advanced computational methods in four metrics (PCC, SSIM, JS, and RMSE) from both gene-level and cell-level.

Additionally, we directly compared the results of [1] with the predictive outcomes of EDGES to further demonstrate its advantages over previous state-of-the-art methods. We evaluated EDGES on the seqFISH_MCA, seqFISH_H, and MERFISH_O datasets, where the PCC-based comparative analysis showed that EDGES ranked second, first, and second, respectively (Table S3). Although EDGES ranked second in two datasets, the differences from the top-performing method, Tangram, were only 0.015 and 0.013, suggesting that EDGES achieves highly competitive predictive performance.

We observed that there are discrepancies between the results of alternative algorithms presented in our result and Table S3, which primarily stem from differences in parameter settings and input data normalization. To more comprehensively evaluate the performance of EDGES, we selected the second-best method, Tangram, as a representative case and conducted replication experiments under different settings. Specifically, we implemented the following two versions: (1) Tangram_norm, in which we adjusted Tangram's parameters to match those in [1] (mode = "clusters") and used normalized expression matrices as input; (2) Tangram_raw, where we used raw count matrices as input while keeping all other settings identical to those in Tangram_norm. Figure S3 shows that EDGES outperforms competing updated methods (Tangram_norm & Tangram_raw) across a range of datasets, maintaining its generally superior predictive performance.

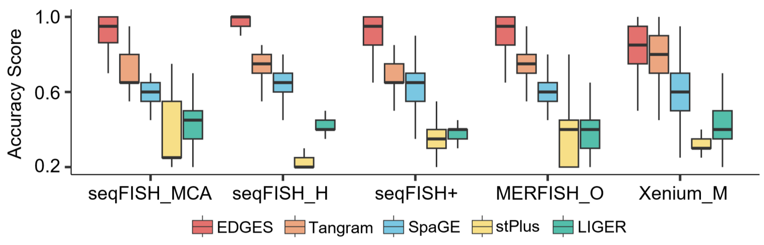

Figure S1. Boxplots show the accuracy scores of each method on five datasets (*n* = 45, 249, 1,000, 1,000, and 541 in each boxplot). The box plot center line and the box limits represent the median value and upper and lower quartiles, respectively. Box whiskers indicate the largest and smallest values no more than 1.5 times the interquartile range from the limits.

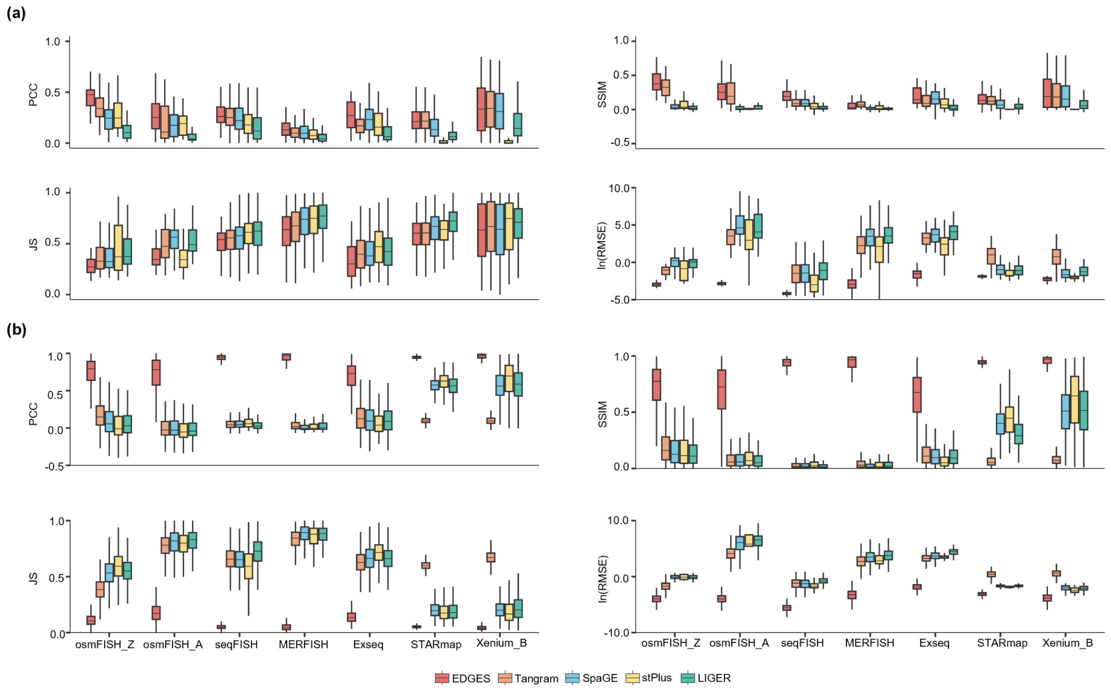

Figure S2. Benchmarking details on validating measured spatial expressions at (a) the gene level (n = 33, 33, 351, 268, 42, 1,020, and 313 in each boxplot) and (b) the cell level (n = the cell number * the fold number in each boxplot). The box plot center line and the box limits represent the median value and upper and lower quartiles, respectively. Box whiskers indicate the largest and smallest values no more than 1.5 times the interquartile range from the limits.

Table S3. Average Pearson correlation coefficients of each method on the seqFISH_MCA, seqFISH_H, and MERFISH_O datasets. The highest predictive performance in each dataset is highlighted in red. Except for EDGES, the results of the other methods were directly obtained from [1].

	seqFISH_MCA	seqFISH_H	MERFISH_O
EDGES	0.3760	0.7429	0.6062
Tangram	0.3911	0.7072	0.6197
SpaGE	0.2512	0.5082	0.5169
stPlus	0.1260	-0.0366	0.0371
LIGER	0	0.0523	0.2079
gimVI	0.2096	0	0.5632
Seurat	0.0811	0.0549	0.3098
SpaOTsc	0.0889	0.0620	0.2300
novoSpaRc	0.1827	0.4542	0.3569

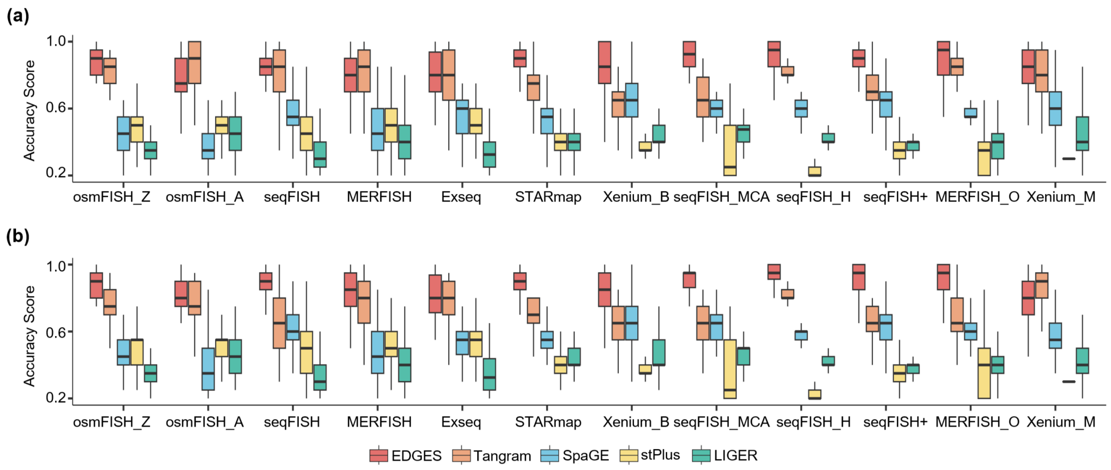

Figure S3. Boxplots show the accuracy scores of each method on twelve datasets (*n* = 33, 33, 351, 268, 42, 1,020, 313, 45, 249, 1,000, 1,000, and 541 in each boxplot). (a) Tangram results generated using mode = 'cluster' and normalized expression matrices as input (Tangram_norm). (b) Tangram results obtained using mode = 'cluster' and raw count matrices as input (Tangram_raw). The box plot center line and the box limits represent the median value and upper and lower quartiles, respectively. Box whiskers indicate the largest and smallest values no more than 1.5 times the interquartile range from the limits.

6. Supporting results for main Figure 3

In this section, we offered supporting materials for the analysis results of EDGES on predicting undetected genes as supplements to Figure 3 in the main text.

Figure S4 shows the number of differentially expressed genes (DEGs) between a specific cell type and other cell types. We found that EDGES increased the number of DEGs across most cell types.

Figure S5 illustrates the functional enrichment of upregulated DEGs specific to astrocytes, excitatory neurons, and ventricular cells. Excitatory neurons-specific DEGs were associated with potassium ion transport and neuronal maturation, processes essential for action potential propagation. In ventricular cells, upregulated DEGs were linked to immune-neural crosstalk and morphogenesis, suggesting a potential role in cardiomyocyte activation and development. Additionally, the spatial distribution of astrocytes-specific upregulated DEGs closely aligned with their corresponding regions, further validating the biological relevance of EDGES predictions.

Figure S6 presents the clustering performance of different methods using Uniform Manifold Approximation and Projection (UMAP). Among them, EDGES generated clustering results that most accurately reflected the actual spatial distribution of cells, demonstrating its superior ability to preserve spatial organization.

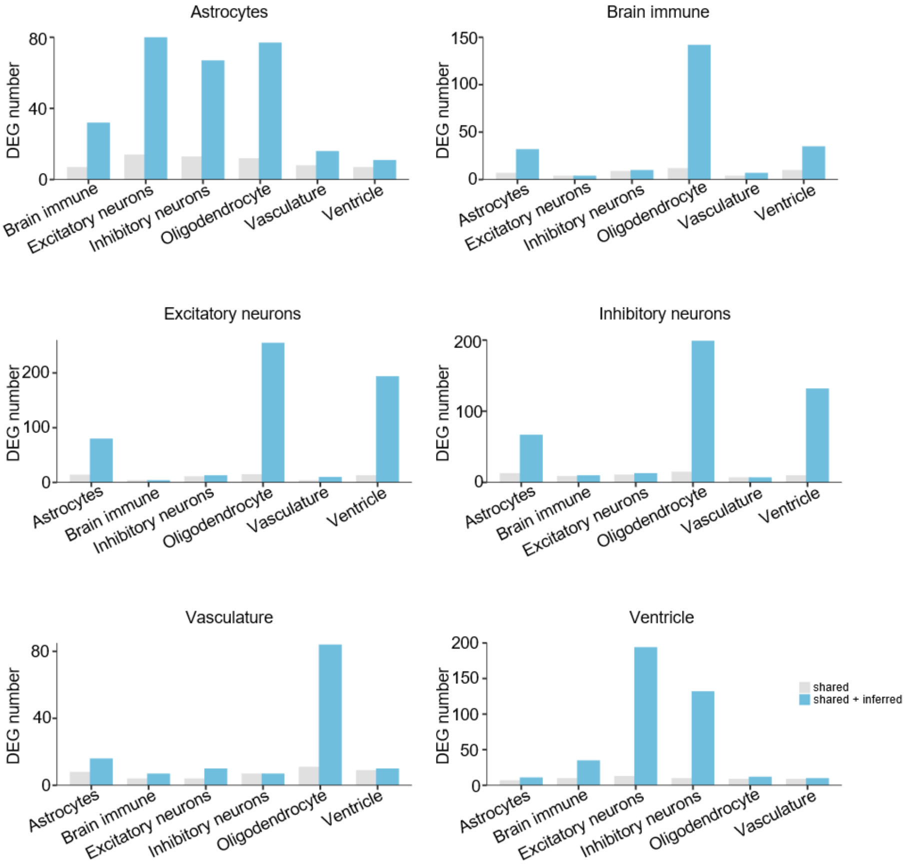

Figure S4. Barplots show the number of DEGs between a specific cell type and other cell types identified from raw data and EDGES-processed data.

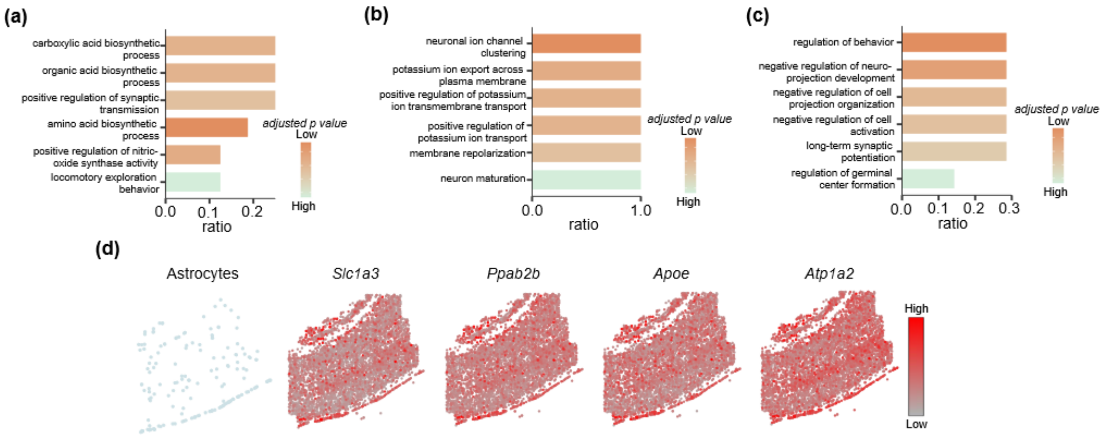

Figure S5. Barplots show the functional enrichment of upregulated DEGs specific to (a) astrocytes, (b) excitatory neurons, and (c) ventricular cells. (d) Visualizations of upregulated DEGs specific to astrocytes.

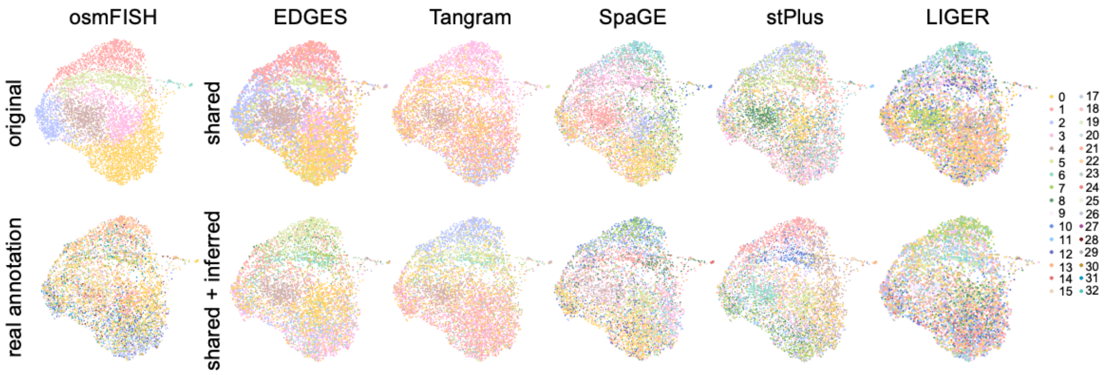

Figure S6. UMAP visualizations of clustering results by different methods using the shared genes (top) and all genes (bottom).

7. Supporting results for main Figure 4

In this section, we presented supporting results demonstrating the denoising performance of EDGES. Figure S7 compares Moran's *I* for the identical spatially variable gene (SVG) across denoised matrices generated by different methods. Our analysis revealed that SVGs identified from the EDGES-denoised data exhibited significantly higher spatial coherence than those obtained from other methods.

To validate the robustness of EDGES's denoising performance, we followed the same analysis pipeline but used SPARK to identify SVGs. As expected, SVGs identified from the EDGES-denoised data exhibited higher Moran's *I* scores. Furthermore, visualizations of SVGs detected by all methods revealed that the EDGES-denoised data displayed more distinct spatial patterns, further underscoring the robustness of EDGES (Figure S8).

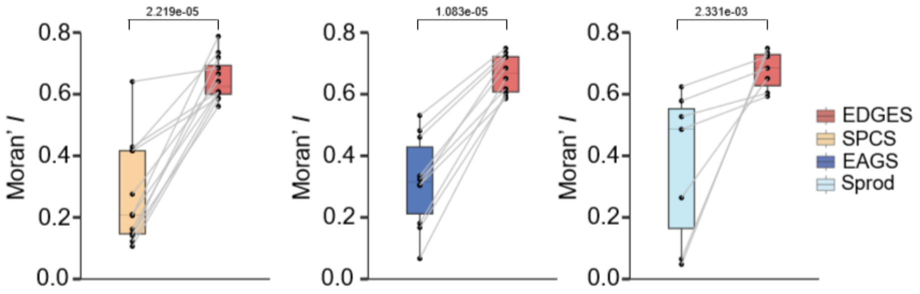

Figure S7. Barplots show the quality of the identical SVGs using the denoised matrices generated by different denoising methods (*n* = 12, 10, 7 in each boxplot). The box plot center line and the box limits represent the median value and upper and lower quartiles, respectively. Box whiskers indicate the largest and smallest values no more than 1.5 times the interquartile range from the limits. The statistical *p* values were determined by the Wilcoxon rank-sum test.

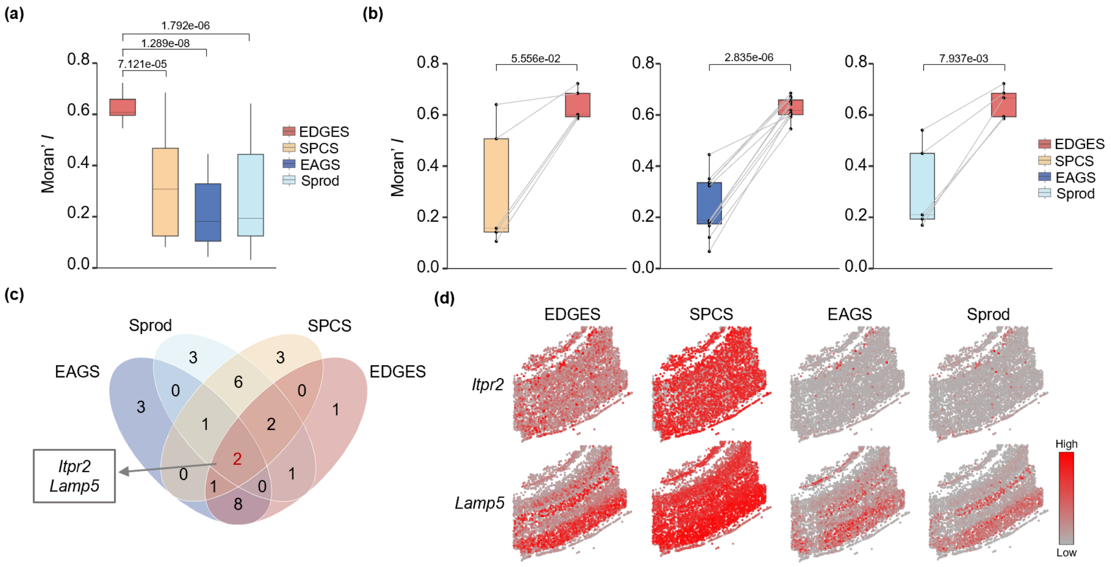

Figure S8. Boxplot shows the quality of (a) the top identified SVGs (n = 15 in each boxplot) and (b) the identical SVGs using the denoised matrices generated by different denoising methods (n = 5, 11, 5 in each boxplot). The box plot center line and the box limits represent the median value and upper and lower quartiles, respectively. Box whiskers indicate the largest and smallest values no more than 1.5 times the interquartile range from the limits. The statistical p values were determined by the Wilcoxon rank-sum test. (c) Venn diagram shows the overlapped SVGs across different denoising methods. (d) Visualizations of specific SVGs with the corresponding expressions denoised by different denoising methods.

8. Supporting results for main Figure 5

In this section, we provided some results to support that EDGES advanced spatial proteomics across applications in human bone marrow.

Figure S9 shows that EDGES enhances the total number of marker proteins distinguishing erythrocytes from other cell types. Interestingly, no differences had previously been detected between erythrocytes and macrophages or monocytes. However, after executing EDGES, the novel marker proteins, such as PLA2G4D for macrophages and monocytes, were identified, effectively distinguishing these cell types.

In the raw data, only CD34 and GYPC were identified as marker proteins for HSPCs and erythrocytes. EDGES expanded this to 20 HSPC-specific and 8 erythrocyte-specific marker proteins (Figure S9). Besides, the spatial distributions of the newly identified marker proteins closely aligned with the localization of their respective cell types (Figure S10).

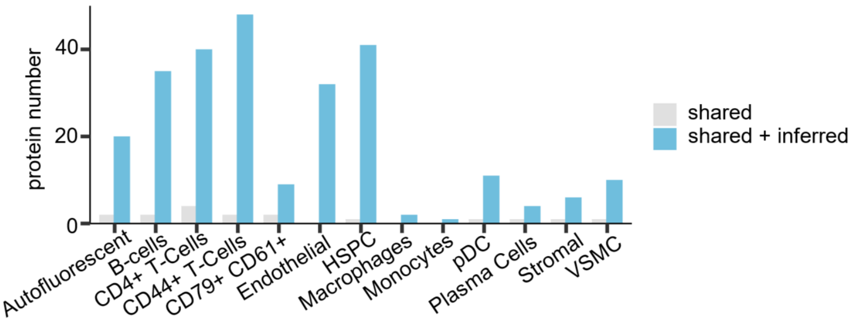

Figure S9. Barplot shows the number of marker proteins between erythrocytes and other cell types identified from raw data and EDGES-processed data.

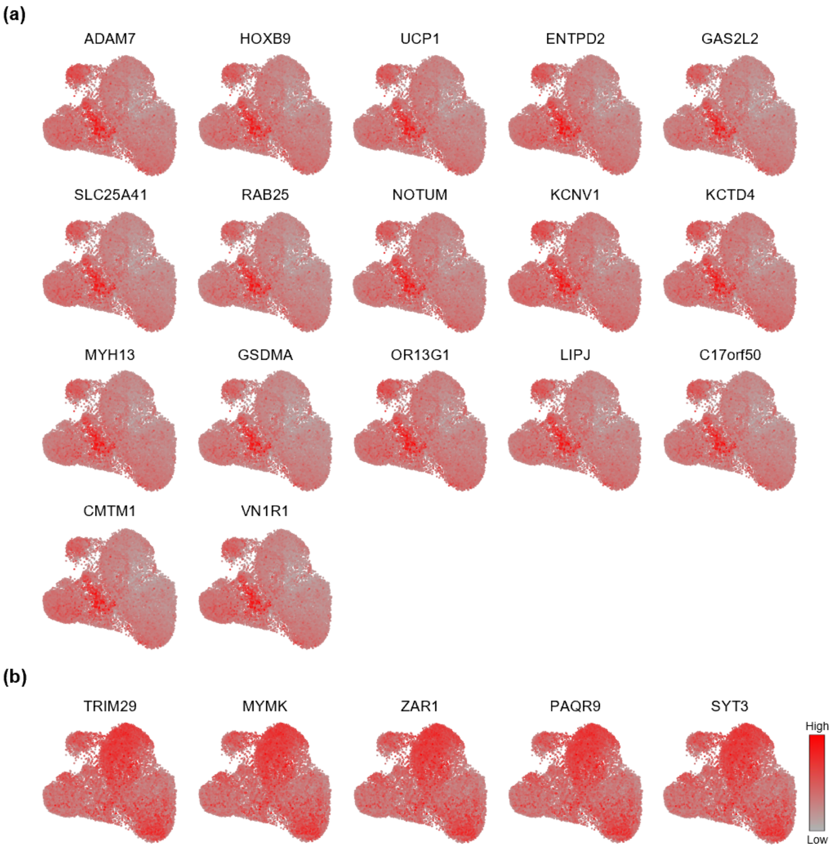

Figure S10. UMAP visualizations of selected marker proteins for (a) HSPCs and (b) erythrocytes, respectively.

9. Supporting results for main Figure 6

In this section, we provided additional results for the BaristaSeq mouse primary visual cortex dataset. Figure S11 shows EDGES enhances the specificity of the marker gene *Dcn* of VISp_I. Figure S12 shows EDGES enhances the specificity of marker genes for each layer. Figure S13 visualizes the expressions of Patterns A and B in the original data, as well as the newly identified Patterns 4 and 5.

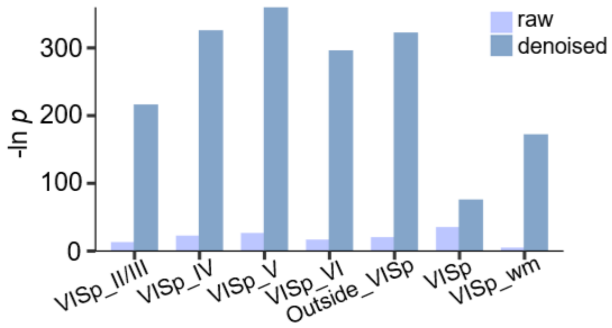

Figure S11. Barplot shows the statistical *p* values of marker genes *Dcn* between VISp_I and other layers before and after EDGES denoising.

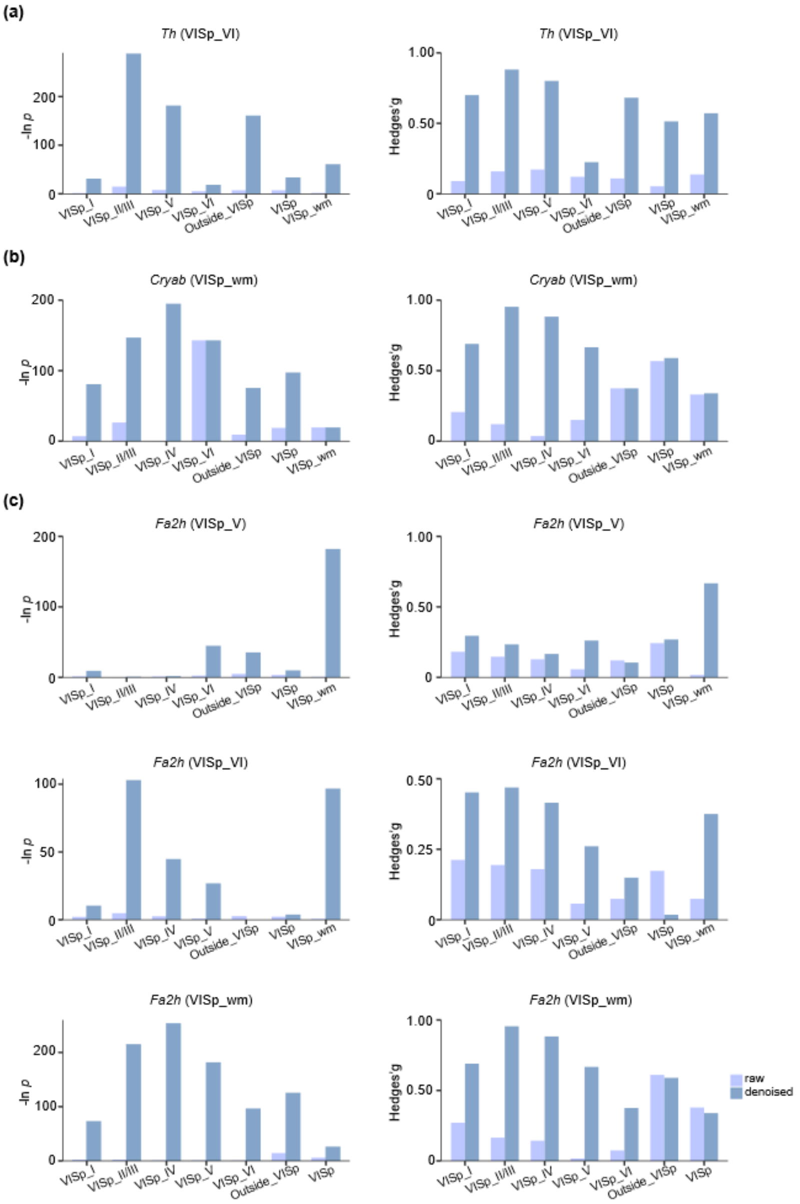

Figure S12. Barplots show the statistical *p* values and Hedges'g values of marker genes between specific layers and other layers before and after EDGES denoising. (a) *Th* for VISP_V. (b) *Cryab* for VISp_wm . (c) *Fa2h* for VISp_V, VISp_VI, and VISp_wm.

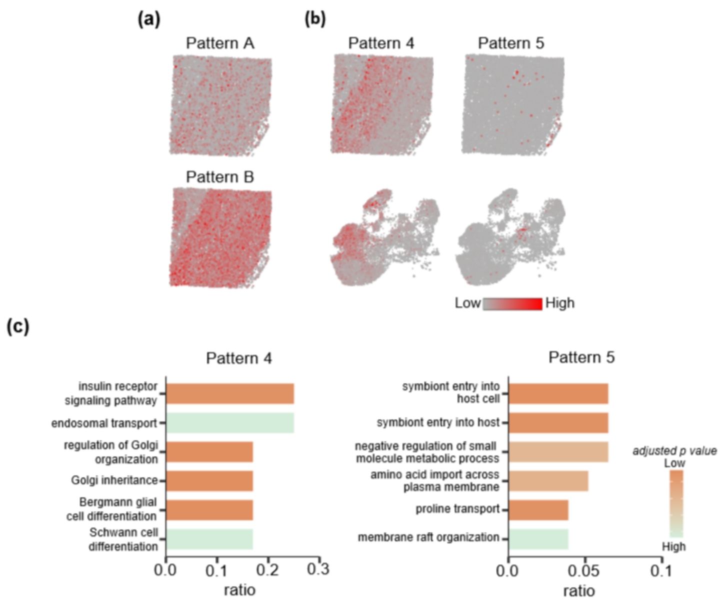

Figure S13. (a) Visualizations of Patterns A and B. (b) Visualizations of newly identified Patterns 4 and 5, with average gene expression (top) and UMAP (bottom). (c) Barplots show the functional enrichment of inferred genes specific to Patterns 4 and 5.

10. Comparison between EDGES with a KNN-based approach

In this section, we assessed the predictive performance of EDGES by comparing it with a *k*-nearest neighbors-based approach (EDGES-KNN), using osmFISH spatial transcriptomics (ST) data and Zeisel's reference scRNA-seq dataset (osmFISH_Z). For each ST cell, EDGES-KNN identified its *K* nearest neighbors (*K* = 10) among the scRNA-seq cells based on their corresponding low-dimensional representations in H_1 and H_2. The average expression profile of these *K* nearest neighbors was then used to predict expression levels of undetected genes in the ST data.

Figure S14 illustrates the comparison results between EDGES and EDGES-KNN. The results show that EDGES significantly outperformed the KNN-based approach at both the gene and cell levels.

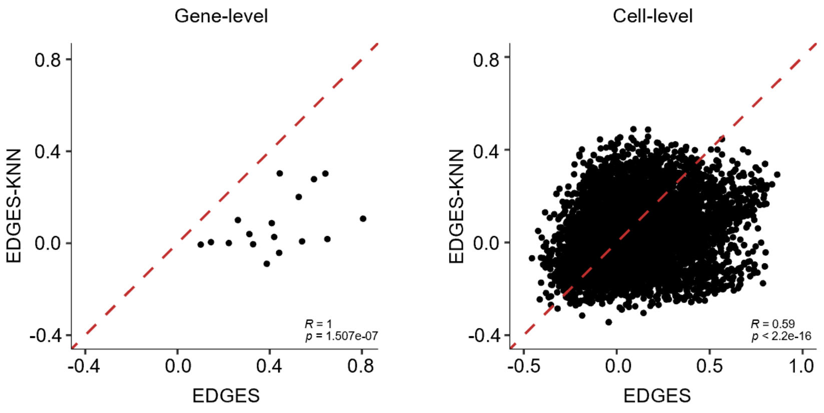

Figure S14. Scatter plots show the predictive performance comparisons between EDGES and EDGES-KNN (*n* = 33 and 6,471 in each plot, respectively). *R* is the proportion of genes with higher PCC in EDGES and the statistical *p* values were determined by the Wilcoxon rank-sum test.

11. Computational efficiency

Table S7. Running time of each method on the selected datasets.

Datasets	Time (s)				
	EDGES	Tangram	SpaGE	stPlus	LIGER
osmFISH_Z	14.09	48.84	32.17	427.64	95.48
osmFISH_A	34.05	30.97	29.79	1161.60	95.23
MERFISH	16.09	181.03	15.69	86.43	65.77
Exseq	8.38	9.86	5.28	39.12	52.39
seqFISH	306.43	6903.86	393.23	3838.83	3838.83

12. Selection of the latent dimension d in EDGES

To justify the choice of latent dimension d in EDGES, we conducted a sensitivity analysis by varying d from 5 to 40 and evaluated the model's predictive performance across this range. By performing cross-validation experiments on osmFISH data, we found that EDGES maintains a robust performance within the range of 15 to 40, with optimal results observed at $d = 20$ (Figure S15). These observations suggest that $d = 20$ strikes a good balance between model complexity and prediction accuracy, while preserving biologically meaningful patterns. Therefore, we set $d = 20$ as the default latent dimension in our study.

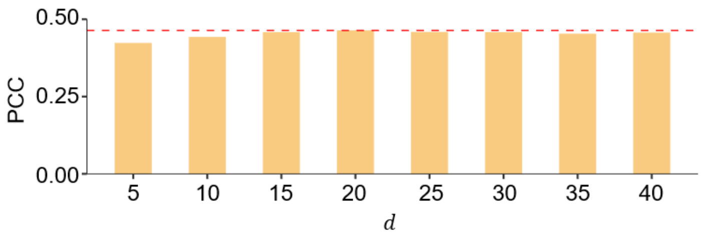

Figure S15. Predictive performance of EDGES using different values of latent dimension d.

13. Hyper-parameter selection of EDGES

In this section, we systematically evaluated the robustness of hyper-parameter selection on the predictive performance using grid search. Predictive performance was quantified using PCC by conducting cross validation on the osmFISH_Z dataset. The default hyper-parameter configuration ($\lambda_1 = 10^{-5},\ \lambda_2 = 10,\ \theta_1 = 10^{-1},\ \theta_2 = 10^{-4}$) achieved optimal gene expression predictive performance (Figure S16). Based on our analysis, we further recommend the following choices for hyper-parameters:

1. Regularization hyper-parameters:
    - λ_1 (spatial regularization): We recommend an operational range of 10^{-7} to 10^{-3}. Values exceeding 10^{0} led to solution divergence.
    - λ_2 (sparsity regularization): Values in the range of 10^{-5} to 10^{3} provided stable computational performance. However, values exceeding 10^{3} resulted in numerical instability.
2. Scaling hyper-parameters:
   We empirically determined the optimal parameter ranges as $\theta_1 \in [10^{-2},\ 10^{2}]$ and $\theta_2 \in [10^{-7},\ 10^{-4}]$, ensuring balanced scaling of the decomposed matrix factors to mitigate numerical discrepancies during optimization.

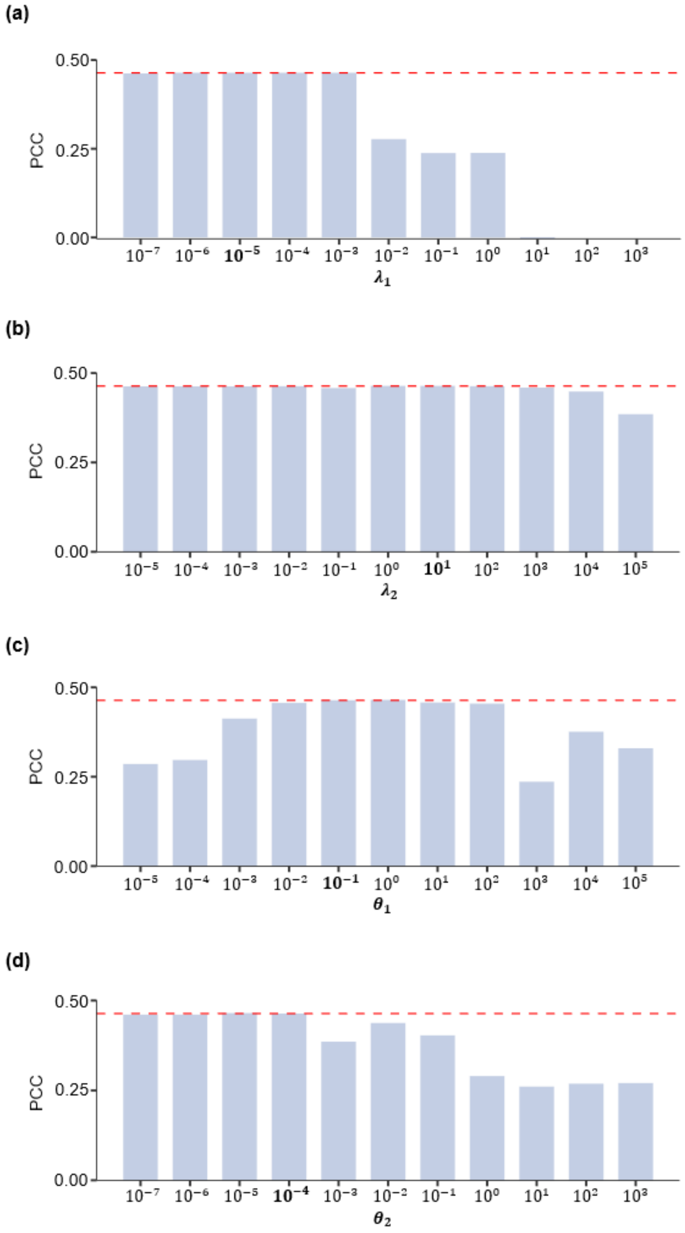

Figure S16. Predictive performance of EDGES using different values of hyper-parameters: (a) λ_1, (b) λ_2, (c) θ_1, and (d) θ_2.

Reference

[1] Li, B., et al., *Benchmarking spatial and single-cell transcriptomics integration methods for transcript distribution prediction and cell type deconvolution.* Nature Methods, 2022. **19**(6): p. 662-670.
